# The Interplay between Retinal Pathways of Cholesterol Output and Its Effects on Mouse Retina

**DOI:** 10.3390/biom9120867

**Published:** 2019-12-12

**Authors:** Alexey M. Petrov, Artem A. Astafev, Natalia Mast, Aicha Saadane, Nicole El-Darzi, Irina A. Pikuleva

**Affiliations:** Department of Ophthalmology and Visual Sciences, Case Western Reserve University, Cleveland, OH 44106, USA; amp280@case.edu (A.M.P.); aastafev93@gmail.com (A.A.A.); nvm2@case.edu (N.M.); asaadane@uci.edu (A.S.); nae20@case.edu (N.E.-D.)

**Keywords:** CYP27A1, CYP46A1, SOAT1, APOE, cholesterol, oxysterols, vesicular traffic, glucose metabolism, fatty acid oxidation, synaptic function, inflammation, photoreceptors, cilia, age-related macular degeneration

## Abstract

In mammalian retina, cholesterol excess is mainly metabolized to oxysterols by cytochromes P450 27A1 (CYP27A1) and 46A1 (CYP46A1) or removed on lipoprotein particles containing apolipoprotein E (APOE). In contrast, esterification by sterol-O-acyltransferase 1 (SOAT) plays only a minor role in this process. Accordingly, retinal cholesterol levels are unchanged in Soat1^−/−^ mice but are increased in Cyp27a1^−/−^Cyp46a1^−/−^ and Apoe^−/−^ mice. Herein, we characterized Cyp27a1^−/−^Cyp46a1^−/−^Soat1^−/−^ and Cyp27a1^−/−^Cyp46a1^−/−^Apoe^−/−^ mice. In the former, retinal cholesterol levels, anatomical gross structure, and vasculature were normal, yet the electroretinographic responses were impaired. Conversely, in Cyp27a1^−/−^Cyp46a1^−/−^Apoe^−/−^ mice, retinal cholesterol levels were increased while anatomical structure and vasculature were unaffected with only male mice showing a decrease in electroretinographic responses. Sterol profiling, qRT-PCR, proteomics, and transmission electron microscopy mapped potential compensatory mechanisms in the Cyp27a1^−/−^Cyp46a1^−/−^Soat1^−/−^ and Cyp27a1^−/−^Cyp46a1^−/−^Apoe^−/−^ retina. These included decreased cholesterol biosynthesis along with enhanced formation of intra- and extracellular vesicles, possibly a reserve mechanism for lowering retinal cholesterol. In addition, there was altered abundance of proteins in Cyp27a1^−/−^Cyp46a1^−/−^Soat1^−/−^ mice that can affect photoreceptor function, survival, and retinal energy homeostasis (glucose and fatty acid metabolism). Therefore, the levels of retinal cholesterol do not seem to predict retinal abnormalities, and it is rather the network of compensatory mechanisms that appears to determine retinal phenotype.

## 1. Introduction

Cholesterol is abundant in mammalian retina, a multilayered light-sensitive tissue lining the back of the eye. The neurosensory retina is underlined by the retinal pigment epithelium (RPE) and then choroid, which nourish the retina. Evidence suggests that retinal cholesterol dyshomeostasis is linked to age-related macular degeneration (AMD), a cause of vision loss in ~200 million older adults worldwide [1]. First, in aging humans, cholesterol accumulates on either side of the RPE and represents a significant component of drusen and subretinal drusenoid deposits [2,3,4,5,6], the AMD hallmarks. Second, genetic studies identified several cholesterol-related genes (*CETP*, *ABCA1*, *LIPC, LPL, VLDLR, LRP6,* and *APOE,* which encodes apolipoprotein E) as risk factors for AMD and suggested that these gene effects are independent of plasma lipid profiles [7,8,9,10,11]. Lastly, the two prospective clinical trials of statins, drugs prescribed for reduction of plasma cholesterol, showed a decreased risk of progression of non-advanced AMD in specific patient populations [12,13]. Moreover, evidence was obtained that intensive statin therapy may even remove drusen in early stages of AMD [13]. These results underscore the importance of understanding the mechanisms by which retinal cholesterol is managed.

**Figure 1:**
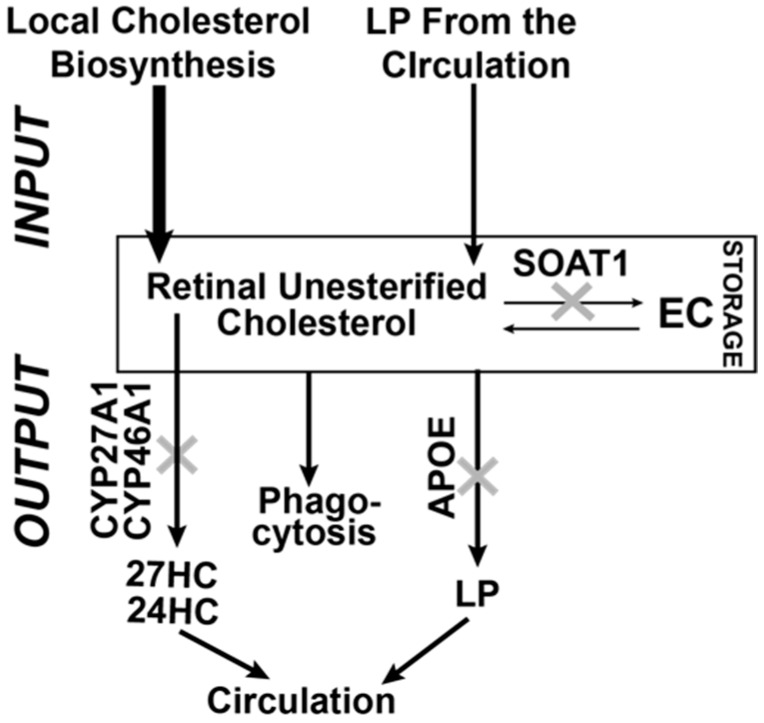
Schematic representation of the known cholesterol-related pathways contributing to cholesterol homeostasis in the retina. Local biosynthesis accounts for the majority (~72%) of retinal cholesterol input, at least in mice, with the remaining cholesterol (~28%) being taken up by the retina from the systemic circulation [23]. A small fraction of retinal cholesterol (up to 15%) is esterified by SOAT1 [24,25] and is stored in the form of lipid droplets. Retinal cholesterol output includes metabolism by CYP27A1 and CYP46A1, the photoreceptor phagocytosis, and removal on lipoprotein particles containing APOE. The relative quantitative contribution of these three pathways to the total cholesterol output in the retina is currently unknown. 27HC and 24HC are 27-hydroxycholesterol and 24-hydroxycholesterol, respectively, the products of CYP27A1 and CYP46A1; EC, esterified cholesterol, the product of SOAT1; LP, lipoprotein particles.

The major pathways of cholesterol input and output in the retina seem to be identified (Figure 1) [14,15,16,17,18,19,20]. Input is dependent on cholesterol biosynthesis in different retinal cells and uptake from the systemic circulation; the latter is likely from the choriocapillaris, as cholesterol delivery from the inner retinal blood vessels is currently speculative [20]. Output includes metabolism to oxysterols by the cytochrome P450 enzymes CYP27A1 and CYP46A1, the photoreceptor phagocytosis, and efflux to lipoprotein particles (LP), which circulate in the intraretinal space. LP then deliver cholesterol to different retinal cells as well as the systemic circulation, probably through the RPE [14,15,16,17,18,19,20]. Intracellular esterification by sterol-O-acyltransferase 1 (SOAT1, named previously as acyl-CoA cholesterol acyltransferase 1 or ACAT1, Text S1) represents the means of cholesterol storage and a form of cholesterol output. Cholesterol esters form lipid droplets, cellular organelles, which provide a unique separation of the aqueous and organic phases of the cell [21]. Cholesterol esters are found in the photoreceptors outer segments, drusen, and Bruch’s membrane [2,3,22].

To ascertain retinal significance of cholesterol removal by metabolism and LP, we previously comprehensively characterized the retina of Cyp27a1^−/−^Cyp46a1^−/−^ mice and mice lacking APOE, a major apolipoprotein in the retina [22,26,27]. In the former, total cholesterol was increased up to 2-fold, and all of the cholesterol surplus was converted to cholesterol esters, which were mainly accumulated in the photoreceptor outer segments [22]. In addition, there were focal accumulations of free (unesterified) cholesterol in the Cyp27a1^−/−^Cyp46a1^−/−^ retina, impairments in the retinal function (as assessed by electroretinographic recordings, ERG), and pathologic changes in retinal blood vessels [26]. In *Apoe^−/−^* mice, retinal cholesterol was also increased (up to 3-fold). However, cholesterol excess remained mainly unesterified and did not form focal deposits as it was mostly present outside retinal cells on the LP circulating in the intraretinal space [27]. Remarkably, the Apoe^−/−^ retina did not have any abnormalities of clinical relevance in structure or in blood vessels, likely because of the compensatory responses triggered by APOE absence. These responses were identified and included changes in the retinal cholesterol biosynthesis and abundance of proteins involved in cellular cytoskeleton maintenance, vesicular traffic, pro- and anti-inflammatory signaling, as well as iron homeostasis [27]. We also generated Cyp27a1^−/−^Cyp46a1^−/−^Soat1^−/−^ mice and established that retinal cholesterol esterification is mediated by SOAT1 [22]. While this genotype had normal levels of retinal cholesterol, signs of retinal degeneration were seen in 6-month old animals—a shortened length of the photoreceptor outer segments as well as increased apoptosis in the photoreceptors [22]. The purpose of the present work was to gain insight into the interplay between retinal pathways of cholesterol output by finishing ophthalmic characterizations of Cyp27a1^−/−^Cyp46a1^−/−^Soat1^−/−^ mice and generating as well as characterizing Cyp27a1^−/−^Cyp46a1^−/−^Apoe^−/−^ mice. We obtained evidence supporting the activation of a previously unrecognized vesicle-mediated mechanism for retinal cholesterol removal in both triple knockouts and suggest that this mechanism serves as a reserve means of retinal cholesterol maintenance. In addition, we mapped retinal proteins and processes in Cyp27a1^−/−^ Cyp46a1^−/−^ Soat1^−/–^ mice that could be linked to retinal cholesterol-related pathways and be important for retinal structure, function, and metabolism.

## 2. Materials and Methods

### 2.1. Animals

Both female and male mice were used, which were 6–12 month old. All animals were free of the Crbl^rd8^ mutation. C57BL/6J and Apoe^−/−^ mice on the C57BL/6J background were from the Jackson Laboratory (catalog nos. 000664 and 002052, respectively). Soat1^−/−^ mice were from the laboratory of Dr. T.Y. Chang (Dartmouth College, Hanover, NH, USA) [28] and were rederived on the C57BL/6J background. Cyp27a1^−/−^ Cyp46a1^−/−^ mice were generated previously in this laboratory [26] and were on a mixed strain background (C57BL/6J; 129S6/SvEv). Cyp27a1^−/−^Cyp46a1^−/−^ mice were crossed with Soat1^−/−^ or Apoe^−/−^ mice to generate Cyp27a1^−/−^Cyp46a1^−/−^Soat1^−/−^ and Cyp27a1^−/−^Cyp46a1^−/−^Apoe^−/−^ mice, respectively. All animals were maintained on a standard 12-h light (~10 lux)-dark cycle and were provided regular rodent chow and water ad libitum. All animal experiments were approved by Case Western Reserve University’s IACUC and conformed to recommendations of the American Veterinary Association Panel on Euthanasia (ethical approval number 2014-0154).

### 2.2. In Vivo Characterizations

Ultra-high resolution spectral-domain optical coherence tomography (SD-OCT), fluorescein angiography (FA), and ERG were carried out as described [26,29]. We used an 840HHP SD-OCT system (Bioptigen, Durham, NC, USA), a scanning laser ophthalmoscope (Spectralis HRA+OCT, Heidelberg Engineering, Heidelberg, Germany), and an Electrophysiological System UTAS E-3000 (LKC Technologies Inc., Gaithersburg, MD, USA), respectively. Images for FA were acquired after a bolus (0.1 mL) intraperitoneal injection of 1.0% sodium fluorescein (Akorn, Lake Forest, IL, USA) in phosphate-buffered saline.

### 2.3. Retinal Sterol Content and Gene Expression

Retinal isolation and processing for sterol quantifications were as described [25,26]. Sterol content (total and unesterified, the former representing a sum of esterified and unesterified cholesterol) was measured in individual retinas by isotope dilution gas chromatography-mass spectroscopy. Deuterated sterol analogs were used as internal standards. For the quantifications of gene expression, retinas from 4–8 mice per genotype were combined, and total RNA was isolated by the TRIzol Reagent (Life Technologies, Grand Island, NY, USA) according to the manufacturer’s instructions. Quantitative RT-PCRs (qRT-PCR) were performed as described [26,30] in triplicate on an LightCycler 480 instrument (Roche Life Science, Penzberg, Germany) using cDNA (obtained from total RNA by SuperScript Reverse Transcriptase, Invitrogen Carlsbad, CA, USA), a pair of gene-specific primers (Table S1), and a FastStart Universal SYBR Green Master (Rox) (Roche Life Science); β-actin served as a reference gene. Changes in relative mRNA levels were calculated by the 2^−ΔΔCt^ method [31].

### 2.4. Retinal Proteomics

The label-free approach was used as described [27]. Briefly, three biological replicates per genotype (Cyp27a1^−/−^Cyp46a1^−/−^Soat1^−/−^ or C57BL/6J, 6–8 month old male mice), each containing two retinas from two different mice, were frozen, cryo-pulverized, and lysed with 3% SDS. Samples were then cleaned of detergent, placed into 50 mM Tris-HCl, pH 8.0, containing 8 M urea, and alkylated with iodoacetaminde. The 8 M urea was next diluted to 4 M with 50 mM Tris-HCl, pH 8.0, and samples were concentrated. Proteins (20 μg of total protein) were sequentially digested with lysyl endopeptidase (Wako Chemicals, Richmond, VA, USA) and trypsin, and an equal amount of internal standard (Pierce Retention Time Calibration Mixture 88321, Thermo Fisher Scientific, Waltham, MA, USA) was added to each sample. Digested proteins (400 ng per sample) were analyzed by LC/MS/MS carried out on a LTQ-Orbitrap Velos mass spectrometer (Thermo Fisher Scientific) equipped with a nanoAcquityTM ultra-high-pressure liquid chromatography system (Waters). Blank injections were run after each sample to minimize carryover between samples. Full-scan MS spectra (*m*/*z* 380–1800) were acquired at a resolution of 60,000 at *m*/*z* 400 followed by 20 data-dependent MS/MS scans generated in the ion-trap detector by collision-induced dissociation of the peptide ions. The retention time, peak intensity, and mass accuracy of five peptides from the internal standard were monitored in all samples. The LC/MS/MS raw data were acquired using the Xcalibur software (Thermo Fisher Scientific) and imported into Rosetta Elucidator™ (RosettaCommons, Burlington, VT, USA). The peak list (.dta) files were searched by Mascot (Matrix Science) against the mouse Uniprot database (538,585 sequences). The search results were imported back into Elucidator, and differences in relative protein abundance were calculated by the PEAKS software (Bioinformatics Solutions Inc, Waterloo, ON, Canada) based on unique peptides [32]. Proteins with non-significant changes (*p* ≥ 0.05) in abundance between the genotypes were excluded from the subsequent proteomics analysis, as are the proteins with less than 2 unique peptides/protein and a 1.5-fold change in the relative abundance, even if this change was significant. Protein grouping was based on the protein function described in the literature (Text S1).

### 2.5. Transmission Electron Microscopy

This was carried out using a 1200EX transmission electron microscope (JEOL Ltd. Tokyo, Japan). Tissue fixation was as described [27,33] and included sequential incubations in 0.1 M Na cacodylate buffer, pH 7.4, containing first 3% glutaraldehyde and then 1% OsO_4_, followed by incubations with 1% tannic acid in 0.05 M Na cacodylate, pH 7.4, and 1% para-phenylenediamine in 70% ethanol.

### 2.6. Statistical Analyses

All images are representative of studies in three to five animals per genotype unless otherwise indicated. All quantitative data represent the mean ± SD or the mean ± SEM; the sample size is indicated in each figure or figure legend. Data were analyzed either by a two-tailed, unpaired Student’s t-test, two-way repeated measures ANOVA, two-way ANOVA with Bonferroni correction, or two-way ANOVA with a Dunnett’s multiple comparisons test. The GraphPad Prism software (GraphPad, San Diego, CA, USA) was used. Statistical significance was defined as * *p* ≤ 0.05; ** *p* ≤ 0.01; *** *p* ≤ 0.001.

## 3. Results

### 3.1. In Vivo Characterizations

**Figure 2:**
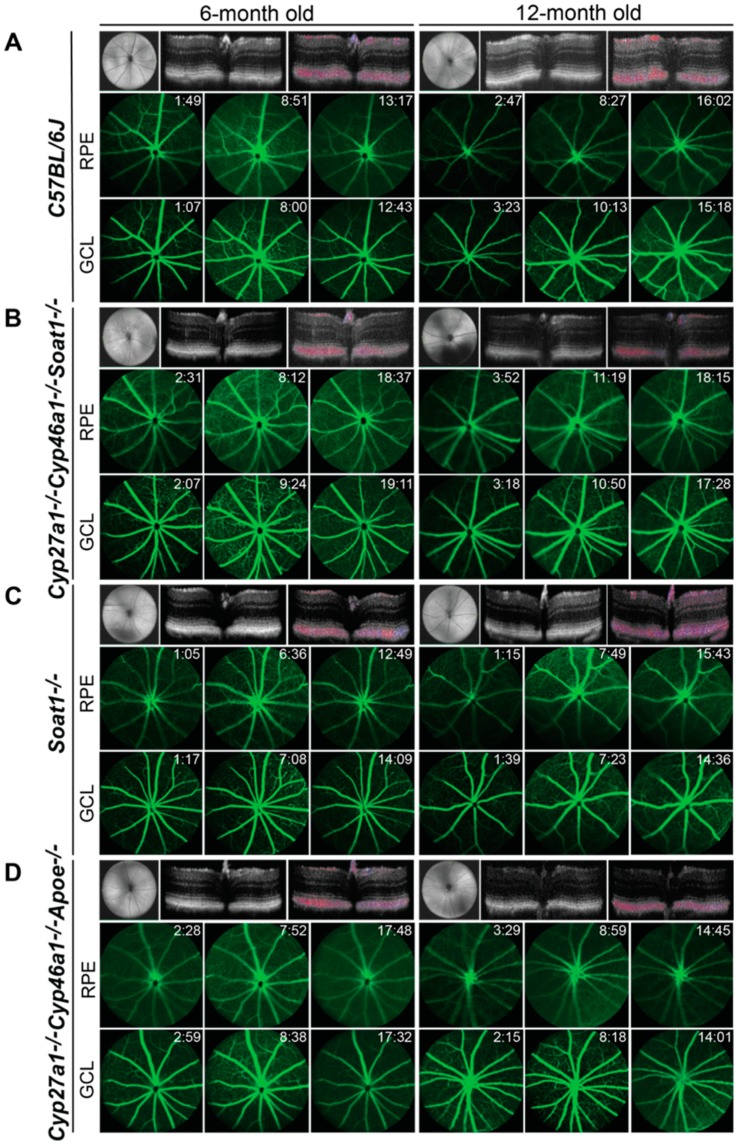
In vivo imaging of 6- and 12-month old C57BL/6J (**A**), Cyp27a1^−/−^Cyp46a1^−/−^Soat1^−/−^ (**B**), Soat1^−/−^ (**C**), and Cyp27a1^−/−^Cyp46a1^−/−^Apoe^−/−^ (**D**) mice. The three rows in each panel are representative images (*n* = 3–5 mice/group) involving SD-OCT (upper panels) and FA (middle and lower panels). The SD-OCT panels show a fundus image and two cross-sections of the retina (from left to right), the latter is a Doppler flow. The FA panels show an early, intermediate, and late stage fundus fluorescence (from left to right) with the laser beam being focused either on the retinal pigment epithelium (RPE) or ganglion cell layer (GCL); the post-injection time is indicated in the top right corner of each image. No sex-based differences were detected, hence only images of male mice are shown.

**Figure 3:**
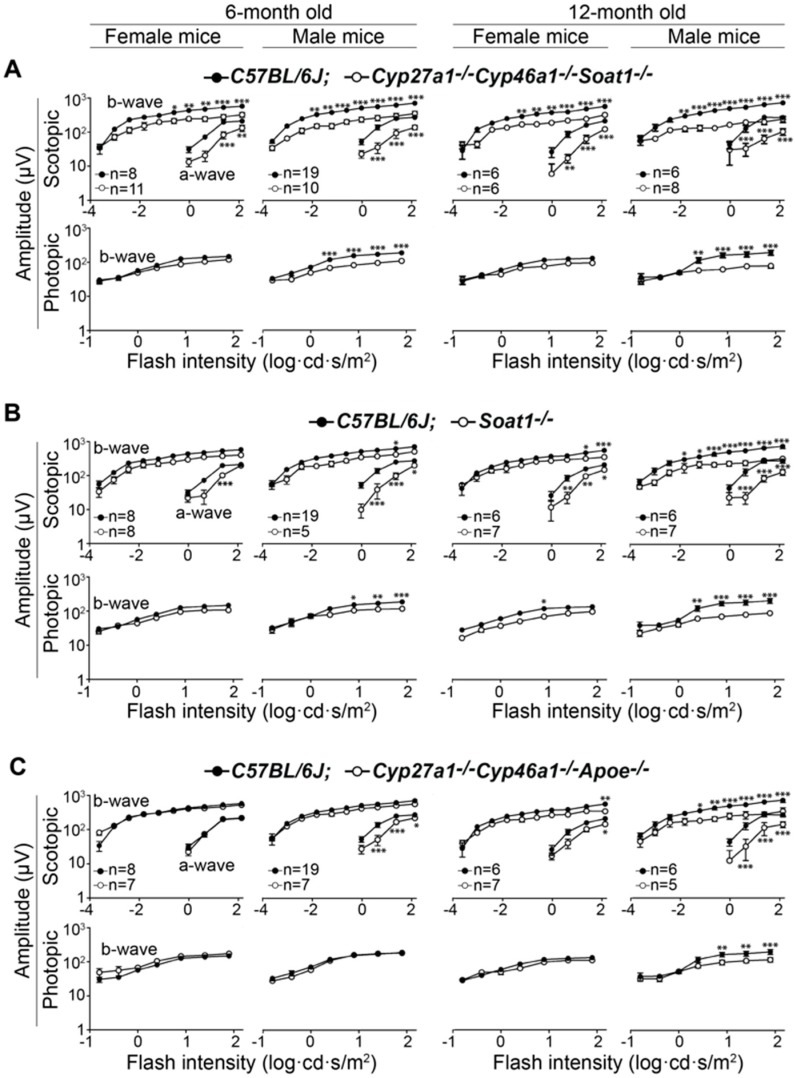
Electroretinography responses in 6- and 12-month old Cyp27a1^−/−^Cyp46a1^−/−^Soat1^−/−^ (**A**), Soat1^−/−^ (**B**), and Cyp27a1^−/−^Cyp46a1^−/−^Apoe^−/−^ (**C**) mice, which were compared with those in C57BL/6J mice. The results are presented as means ± SEM of the measurements in 5–19 animals. * *p* ≤ 0.05; ** *p* ≤ 0.01; *** *p* ≤ 0.001 as assessed by repeated measures two-way ANOVA, where flash intensity was used as a repeated variable and interaction between sex and genotype was evaluated for significance.

We examined 6- and 12- month old Cyp27a1^−/−^Cyp46a1^−/−^Soat1^−/−^ and Cyp27a1^−/−^Cyp46a1^−/−^ Apoe^−/−^ by using SD-OCT, FA, and ERG. We also included studies of Soat1^−/−^ mice as their retinal phenotype has not been reported. None of the three genotypes appeared to have any gross abnormalities in the retinal structure as assessed by SD-OCT (Figure 2). Also, the three genotypes did not have major abnormalities in the retinal blood vessels as evaluated by FA (Figure 2). However, studies by ERG revealed age-dependent changes in overall retinal function. In Cyp27a1^−/−^ Cyp46a1^−/−^ Soat1^−/−^ mice of both sexes, dark-adapted (scotopic) ERGs were decreased at 6 months, and this decrease was more pronounced at 12 months (Figure 3A). In addition, male Cyp27a1^−/−^Cyp46a1^−/−^ Soat1^−/−^ mice had a decrease in the b- wave of light-adapted (photopic) ERGs, also with a progression at 1 year. In Soat1^−/−^ mice, ERGs were decreased as well, especially in 12-month old males, suggesting that lack of the SOAT1-mediated cholesterol esterification can affect retinal function. In Cyp27a1^−/−^ Cyp46a1^−/−^Apoe^−/−^ mice, the ERGs of female animals were essentially unaffected, and only male mice had statistically significant reductions in both scotopic and photopic ERGs at 12 months (Figure 3C). Thus, in vivo evaluations revealed that the major effect of the two triple gene ablations was on retinal function, but not on retinal structure and vasculature.

### 3.2. Retinal Sterol Content

**Figure 4:**
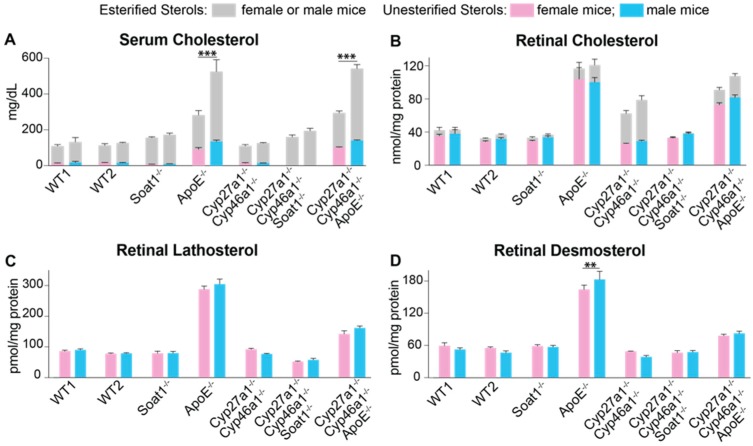
Serum (**A**) and retinal (**B**–**D**) sterols. WT1, wild type mice (C57BL/6J) for the Soat1^−/−^, Apoe^−/−^, Cyp27a1^−/−^Cyp46a1^−/−^Soat1^−/−^, and Cyp27a1^−/−^Cyp46a1^−/−^Apoe^−/−^ genotypes; WT2, wild type mice (C57BL/6J; 129S6/SvEv) for the Cyp27a1^−/−^Cyp46a1^−/−^ genotype. The results are presented as means ± SD of the individual measurements in 3–7 animals/genotype/sex; number of mice equals the number of retinas; animals were 6–8-months old. Statistical significance was assessed by two-way ANOVA with Bonferroni correction. Black lines and asterisks indicate statistically significant differences between sexes of the same genotype; the results of the other comparisons are summarized in Tables S2–S5. ** *p* ≤ 0.01; *** *p* ≤ 0.001.

Three retinal sterols (cholesterol, lathosterol, and desmosterol) as well as serum cholesterol were quantified in Cyp27a1^−/−^Cyp46a1^−/−^Soat1^−/−^, Cyp27a1^−/−^Cyp46a1^−/−^Apoe^−/−^, and Soat1^−/−^ mice and compared with those in other knockout lines determined by us previously [22,26,27]. In both serum and retina, the levels of total cholesterol in Cyp27a1^−/−^Cyp46a1^−/−^Soat1^−/−^ and Cyp27a1^−/−^Cyp46a1^−/−^Apoe^−/−^ mice were more similar to those in Soat1^−/−^ and Apoe^−/−^ mice, respectively, than in Cyp27a1^−/−^Cyp46a1^−/−^ mice (Figure 4A,B, Tables S2 and S3). Yet serum cholesterol did not seem to be an important contributor to retinal cholesterol as the sex differences in the levels of this sterol in Apoe^−/−^ and Cyp27a1^−/−^Cyp46a1^−/−^Apoe^−/−^ mice were not translated into similar sex differences in the levels of total retinal cholesterol. Further, retinal lathosterol and desmosterol levels serve as markers for cholesterol biosynthesis in neurons and astrocytes, respectively [34,35]. In Cyp27a1^−/−^Cyp46a1^−/−^Soat1^−/−^ mice, retinal lathosterol but not desmosterol content was decreased as compared to those in Cyp27a1^−/−^Cyp46a1^−/−^ and Soat1^−/−^ mice (Figure 4C,D, Tables S4 and S5). This compensatory decrease is consistent with the known sensitivity of cholesterol biosynthesis to changes in intracellular levels of unesterified cholesterol [36] and can be a reason for why total retinal cholesterol was normal in Cyp27a1^−/−^Cyp46a1^−/−^Soat1^−/−^ mice. Similarly, a decrease in retinal cholesterol biosynthesis was observed in Cyp27a1^−/−^Cyp46a1^−/−^Apoe^−/−^ mice, whose levels of both retinal lathosterol and desmosterol were approximately the mean between the sterol levels in Apoe^−/−^ and Cyp27a1^−/−^Cyp46a1^−/−^ mice and mirrored the total cholesterol levels in the retina of the three genotypes. Thus, serum and retinal sterol profiling identified a decrease in retinal cholesterol biosynthesis as a compensatory response in both triple knockout genotypes.

### 3.3. Retinal Gene Expression

**Figure 5:**
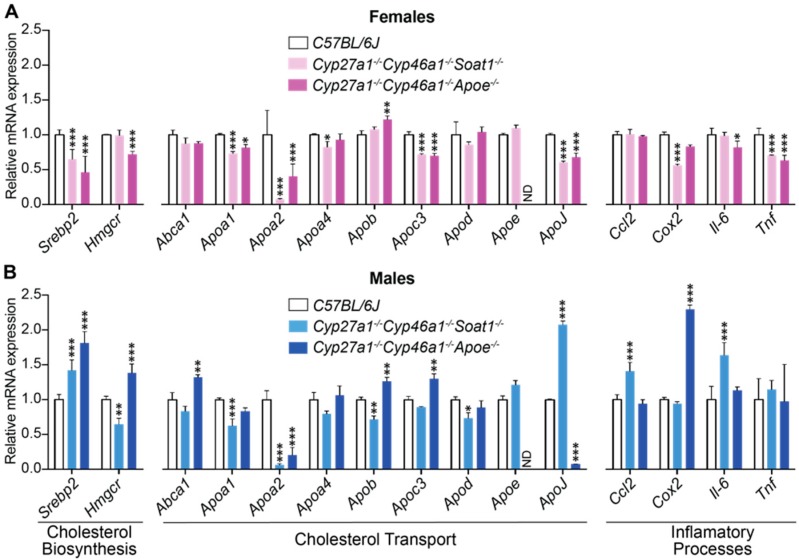
Retinal expression of the genes related to cholesterol and inflammation in female (**A**) and male (**B**) mice. The results are presented as means ± SD of triplicate measurements in pooled samples from 6–8 month old C57BL/6J female mice (*n* = 7 mice, 13 retinas), *C57BL/6J* male mice (*n* = 8 mice, 14 retinas), Cyp27a1^−/−^Cyp46a1^−/−^Soat1^−/−^ female mice (*n* = 5 mice, 10 retinas), Cyp27a1^−/−^Cyp46a1^−/−^Soat1^−/−^ male mice (*n* = 6 mice, 12 retinas), Cyp27a1^−/−^Cyp46a1^−/−^ApoE^−/−^ female mice (*n* = 5 mice, 10 retinas), and Cyp27a1^−/−^Cyp46a1^−/−^ApoE^−/−^ male mice (*n* = 4 mice, 8 retinas). * *p* ≤ 0.05; ** *p* ≤ 0.01; *** *p* ≤ 0.001 vs. the C57BL/6J retina as assessed by two-way ANOVA with the post-hoc Dunnet’s test. ND, not detectable.

Two groups of retinal genes were quantified in Cyp27a1^−/−^Cyp46a1^−/−^Soat1^−/−^ and Cyp27a1^−/−^Cyp46a1^−/−^Apoe^−/−^ mice and compared with that in wild type (WT) C57BL/6J animals (Figure 5). The first included the genes related to cholesterol biosynthesis (*Hmgcr*) and its transcriptional regulation (*Srebp2*) as well as apolipoprotein-mediated cholesterol transport (*Abca1*, *Apoa 1* and *2*, *Apoa4*, *Apob*, *Apoc3*, *Apod*, *Apoe*, and *Apoj*). The second were the genes related to inflammation (*Ccl2*, *Cox2*, *Il-6*, and *Tnfα*), whose expression is controlled by NF-κB, which in turn is negatively regulated by liver X receptors (LXRs, transcription factors) when they are activated by oxysterols produced by CYP27A1 and CYP46A1 [37]. Statistically significant changes were detected in the expression of almost every studied gene. However, these changes were mostly sex-specific and therefore likely did not contribute to retinal cholesterol levels that were similar in female and male animals of the Cyp27a1^−/−^Cyp46a1^−/−^ Soat1^−/−^ and Cyp27a1^−/−^Cyp46a1^−/−^Apoe^−/−^ genotypes. Only *Apoa1* and *Apoa2* in Cyp27a1^−/−^Cyp46a1^−/−^Soat1^−/−^ mice as well as *Apob* and *Apoj* in Cyp27a1^−/−^Cyp46a1^−/−^Apoe^−/−^ mice showed expression changes that are common for both sexes. The ambiguous results of the retinal gene expression justified subsequent use of retinal proteomics to compare the relative protein levels.

### 3.4. Retinal Proteomics

**Figure 6:**
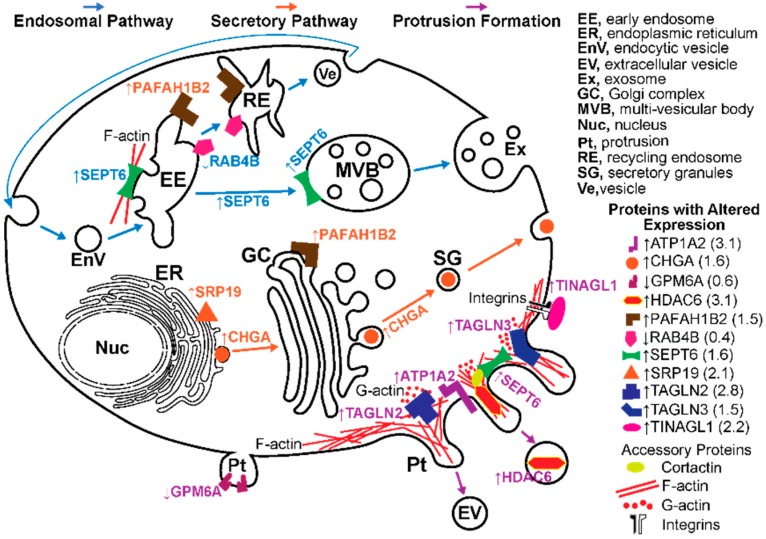
Differentially expressed proteins in the Cyp27a1^−/−^Cyp46a1^−/−^Soat1^−/−^ vs. wild type retina of pertinence to the formation of vesicles and protrusions. Vesicular formation via endosomal pathway is shown in blue font and arrows and that via the secretory pathway is shown in orange font and arrows; the formation of membrane protrusions (Pt) is indicated in purple font and arrows. Protein full names and all the supporting references are provided in the Text S1. In the endosomal pathway, SEPT6 (an F-actin binding protein) is required for the maturation of early endosomes (EE) into multi-vesicular bodies (MVB), whereas RAB4B (a small GTPase) and PAFAH1B2 (an endosome-associated phospholipase) participate in material delivery from EE to recycling endosomes (RE) and subsequently to plasma membrane via vesicles (Ve). Increased expression of SEPT6 and PAFAH1B2 along with a decreased expression of RAB4B point to an increased MVB but not RE formation and therefore an increased exosome (Ex) release. In the secretory pathway, CHGA (a neurosecretory protein) is present in all of the pathway-related organelles: endoplasmic reticulum (ER), Golgi complex (GC), and secretory granules (SG). CHGA expression initiates SG formation and increases a number of SG. SRP19 is responsible for translation and targeting of the membrane and secretory proteins to ER and their further routing via the secretory pathway. When PAFAH1B2 is bound to GC, it maintains GC integrity and therefore the functionality of the secretory pathway. Increased abundance of SRP19, CHGA, and PAFAH1B2 points to an increase in membrane trafficking via the secretory pathway. Lastly, Pt formation involves TAGLN2, TAGLN3, SEPT, and GPM6A6. TAGLN2 and TAGLN3 increase G-actin polymerization. SEPT6 acts on actin assembly indirectly via cortactin, a regulator of actin polymerization and a substrate for HDAC6, which deacetylates cortactin and increases its binding to actin. HDAC6 was found in extracellular vesicles (EV) derived from Pt. GPM6A (a transmembrane protein) induces clustering of lipid rafts and facilitates the Pt formation independent of actin. Pt formation can also be indirectly affected by TINAGL1 (an extracellular matrix protein) and ATP1A2 (a transmembrane protein), which can be associated with the actin cytoskeleton. Increased expression of TAGLN2, TAGLN3, SEPT, TINAGL1, and ATP1A2 raises a possibility that Pt formation could be increased as well and could increase the formation of extracellular vesicles (EV).

**Figure 7:**
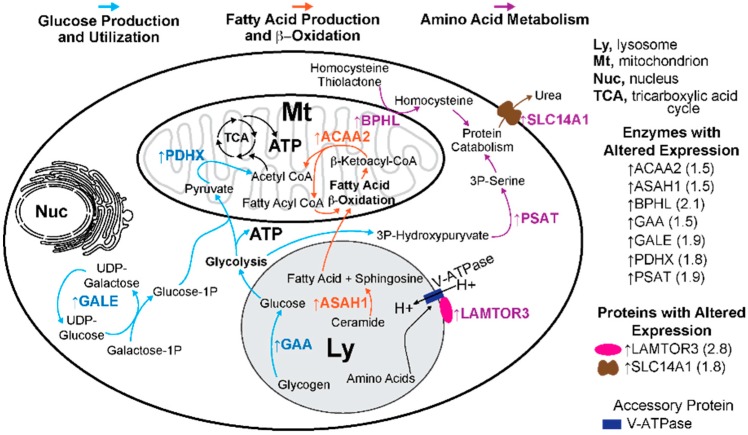
Differentially expressed proteins in the Cyp27a1^−/−^Cyp46a1^−/−^Soat1^−/−^ vs. wild type retina of pertinence to energy and protein homeostasis. Glucose production and utilization are indicated in blue font and arrows; fatty acid production and β-oxidation are in orange font and arrows; and amino acid metabolism is in purple font and arrows. Protein full names and all the supporting references are provided in the Text S1. In glucose production and utilization, GAA (a lysosomal enzyme) catalyzes glucose release from glycogen. Simultaneously, in the cytoplasm, glucose-1-phosphate (1P) is generated by GALE action, which catalyzes the final step in the major pathway of galactose metabolism. Both glucose and glucose-1P are fuel for glycolysis, which produces energy in the form of ATP. Pyruvate, the end product of glycolysis, enters the mitochondria and is converted to acetyl CoA by the PDH complex containing PDHX. This PDH-dependent reaction is the rate limiting step for the utilization of pyruvate under aerobic conditions. Further, in the fatty acid-related pathways, free fatty acids can be released in lysosomes from the breakdown of ceramides by ASAH1 and then reach the mitochondria, where they enter β-oxidation. The last step in β-oxidation is catalyzed by ACAA2 to yield acetyl CoA. Acetyl CoA from both pyruvate and fatty acid β-oxidation enters the tricarboxylic acid cycle (TCA) to produce ATP. Lastly, in amino acid metabolism, the amino acid levels in the lysosomes are sensed by the protein complex including LAMTOR3 and V-ATPase (a proton pump). In the cytoplasm, 3P-hydroxypyruvate (produced from the glycolysis intermediate) is converted to 3P-serine by PSAT1. In the mitochondria, homocysteine thiolactone (a by-product of protein biosynthesis) could be converted to homocysteine by BPHL. Both 3P-serine and homocysteine are the precursors of cysteine used for protein synthesis. Catabolism of proteins produces urea, which can be transported from cells by SLC14A1. The expression of all the enzymes indicated in this figure was increased in the Cyp27a1^−/−^Cyp46a1^−/−^Soat1^−/−^ vs. WT retina, thus raising a possibility of increased energy production as well as altered protein homeostasis.

This was conducted on Cyp27a1^−/−^Cyp46a1^−/−^Soat1^−/−^ vs. WT male mice to gain insight into whether there are additional mechanisms that contribute to the normalization of retinal cholesterol levels besides a decrease in cholesterol biosynthesis. We also sought to map the mechanisms that impair retinal function, when the retinal cholesterol levels are normal. The label-free analysis was used, which confirmed unaltered retinal expression of APOA4 and APOE (Table 1) suggested by the mRNA measurements (Figure 5) but did not show altered abundance of APOA1, APOA2, and APOJ, whose gene expression was affected in the Cyp27a1^−/−^Cyp46a1^−/−^Soat1^−/−^ vs. WT retina of male mice (Figure 5). Proteins encoded by *Srebp2*, *Hmgcr*, *Abca1*, *Apob*, *Apoc3*, and *Apod* were not detected. Nevertheless, collectively, the data obtained suggested that cholesterol transport mediated by the major retinal apolipoproteins did not seem to be significantly affected by simultaneous absence of CYP27A1, CYP46A1, and SOAT1. In addition, we identified 43 proteins with a significantly different abundance. Out of these proteins, the abundance of 41 proteins was increased and that of 3 proteins was decreased in the Cyp27a1^−/−^Cyp46a1^−/−^Soat1^−/−^ vs. WT retina (Table 2). Protein grouping based on function (Text S1) suggested that at least 6 biological processes could be affected in the Cyp27a1^−/−^Cyp46a1^−/−^Soat1^−/−^ retina: (1) cytoskeletal dynamics and vesicle formation (11 proteins, Figure 6); (2) energy homeostasis (9 proteins, Figure 7); (3) genetic information transfer (DNA/RNA processing, transcription, regulation of transcription and translation, 9 proteins); (4) inflammation (7 proteins); (5) synaptic function (4 proteins); and (6) ubiquitination (3 proteins). Since changes in the vesicle formation can be detected by TEM, we evaluated retinal ultrastructure next.

### 3.5. TEM

**Figure 8:**
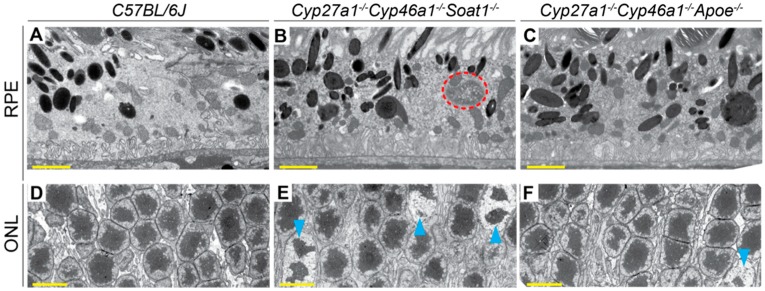
Retinal ultrastructure as assessed by TEM. Cross sections through the retinal pigment epithelium (RPE) and outer nuclear layer (ONL) are shown. Orange dashed ellipse denotes undigested material; cyan arrowheads denote chromatin condensation. **A**, **B**, **D**, **E**: Images are representative of three male mice per genotype. C, F: Only one male animal was imaged. All mice were 7-months old. Scale bars: 2 μm (**A**,**B**,**C**); 5 μm (**D**,**E**,**F**).

**Figure 9:**
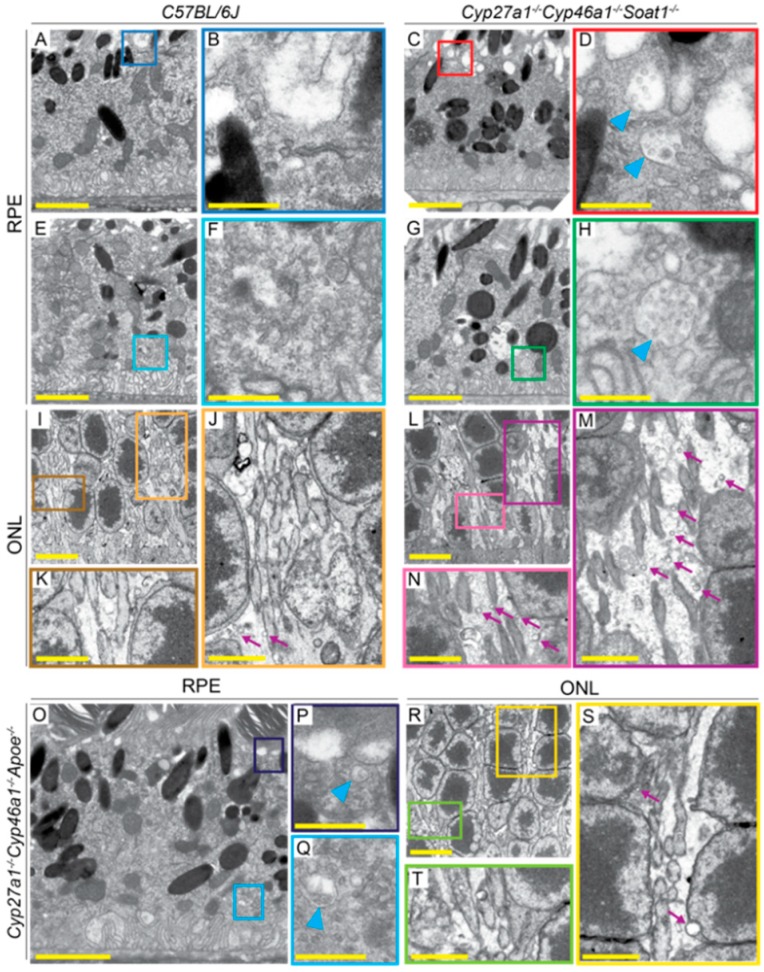
Retinal ultrastructure as assessed by TEM. Colored squares and rectangles in **A**, **C**, **E**, **G**, **I**, **L**, **O**, and **R** denote regions that are shown at a higher magnification in **B**, **D**, **F**, **H**, **J**, **K**, **M**, **N**, **P**, **Q**, **S**, and **T**. Cyan arrowheads denote some of the vesicles in the **RPE**; purple arrows denote some of the vesicles in the extracellular space of the ONL. **A**–**M**: Images are representative of three male mice per genotype. **O**–**T**: Only one male animal was imaged. All mice were 7-months old. Scale bars: 2 μm (**A**,**C**,**E**,**G**,**J**,**K**,**M**,**N**,**O**,**S**,**T**); 5μm (**I**,**L**,**R**); 0.5 μm (**B**,**D**,**F**,**H**,**P**,**Q**).

**Figure 10:**
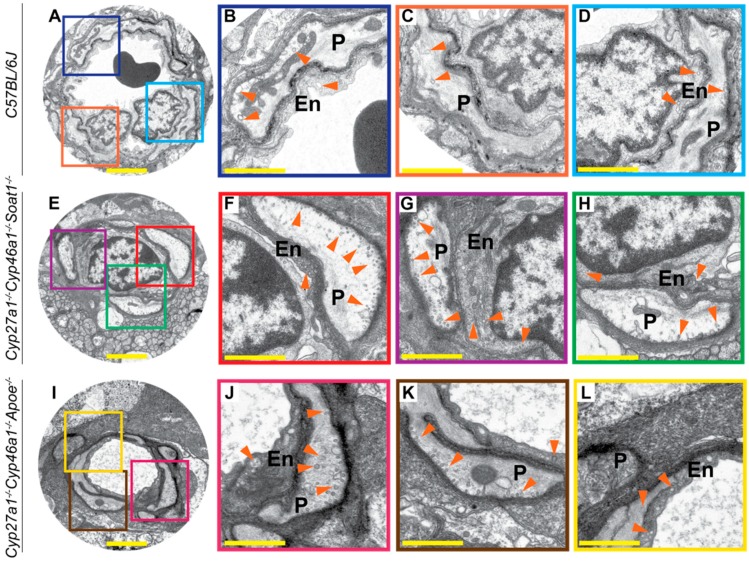
Retinal ultrastructure as assessed by TEM. Colored squares in **A**, **E**, **I** denote regions in the neurovascular units of the ganglion cell layers that are shown at a higher magnification in **B**–**D**, **F**–**H**, **J**–**L**. Orange arrowheads denote some of the vesicles. En, vascular endothelium; P, pericyte. **A**–**H**: Images are representative of three mice per genotype. **I**–**L**: Only one animal was imaged. All mice were 7-month old males. Scale bars: 2 μm (**A**,**E**,**I**); 1 μm (**B**–**D**,**F**–**H**,**J**–**L**).

Postfixation with osmium, tannic acid, and p-phenylenediamine was used, which preserves and enhances the visualization of membranous structures (tannic acid) and osmium-treated neutral lipids (p-phenylenediamine) [33]. This approach revealed several differences between WT, Cyp27a1^−/−^Cyp46a1^−/−^Soat1^−/−^, and Cyp27a1^−/−^Cyp46a1^−/−^Apoe^−/−^ mice. First, undigested material was observed in some of the RPE cells of Cyp27a1^−/−^Cyp46a1^−/−^Soat1^−/−^ mice but not WT or Cyp27a1^−/−^Cyp46a1^−/−^Apoe^−/−^ mice (Figure 8A–C), probably a reflection of impaired phagocytosis at the phagolysosomal stage [38]. Second, chromatin condensation was more abundant in the photoreceptor nuclei of Cyp27a1^−/−^Cyp46a1^−/−^Soat1^−/−^ than Cyp27a1^−/−^Cyp46a1^−/−^Apoe^−/−^ mice and was essentially absent in WT mice (Figure 8D–F). This observation is consistent with increased photoreceptor apoptosis documented previously in Cyp27a1^−/−^Cyp46a1^−/−^Soat1^−/−^ mice [22]. Third, Cyp27a1^−/−^Cyp46a1^−/−^Soat1^−/−^ mice had increased, as compared to WT and Cyp27a1^−/−^Cyp46a1^−/−^Apoe^−/−^ mice, with the presence of multivesicular bodies at both apical and basal sides of the RPE (Figure 9A–H, O–Q) in agreement with the change in vesicular traffic suggested by the proteomics analysis (Figure 6). Similarly, Cyp27a1^−/−^Cyp46a1^−/−^Soat1^−/−^ mice had more frequent extracellular vesicles in the outer nuclear layer (Figure 9I–M, R–T), and extensive budding of the caveoli-like structures in the blood vessel pericytes and endothelium of the ganglion cell layer (Figure 10). Thus, intracellular and extracellular vesicular traffic seemed to be increased in Cyp27a1^−/−^Cyp46a1^−/−^Soat1^−/−^ relative to WT or Cyp27a1^−/−^Cyp46a1^−/−^Apoe^−/−^ mice and directed toward both choriocapillaries and inner retina blood vessels.

## 4. Discussion

In the present work, we continued to investigate retinal mechanisms that maintain cholesterol homeostasis and could be important for retinal structure and function [22,26,27,29,30,39,40]. Only three enzymes (CYP27A1, CYP46A1, and SOAT1) metabolize intracellular (unesterified) cholesterol in the retina (Figure 1) with retinal cholesterol content being almost doubled in Cyp27a1^−/−^Cyp46a1^−/−^ mice and normal in Soat1^−/−^ mice (Figure 4) [22,26]. Hence by generating Cyp27a1^−/−^Cyp46a1^−/−^Soat1^−/−^ mice, we expected that their retinal cholesterol will still be increased as in Cyp27a1^−/−^Cyp46a1^−/−^ mice but be unesterified as in Soat1^−/−^ mice (Figure 4B). Yet, the retinal cholesterol levels were normal in Cyp27a1^−/−^Cyp46a1^−/−^Soat1^−/−^ mice (Figure 4B), thus suggesting that there are compensatory responses in the retina, which lowered the sterol levels. Similarly, by generating Cyp27a1^−/−^Cyp46a1^−/−^Apoe^−/−^ mice, we expected an additive genotype effect due to simultaneous blockage of the two major pathways of retinal cholesterol output - cholesterol removal via metabolism (CYP27A1 and CYP46A1) and LP containing APOE which accepts cholesterol effluxed from cells. However, the Cyp27a1^−/−^Cyp46a1^−/−^Apoe^−/−^ retina had lower, not higher, total cholesterol levels than the Apoe^−/−^ retina (Figure 4B), also an indication of the compensatory responses. Therefore, to map these compensatory responses, we first conducted retinal sterol profiling and quantified cholesterol precursors lathosterol and desmosterol. The levels of lathosterol were decreased in the Cyp27a1^−/−^Cyp46a1^−/−^Soat1^−/−^ retina as compared to Soat1^−/−^ and Cyp27a1^−/−^Cyp46a1^−/−^ retina, and both sterols were decreased in the Cyp27a1^−/−^Cyp46a1^−/−^Apoe^−/−^ retina as compared to the Apoe^−/−^ retina (Figure 4C,D). These decreases suggested that the retinal cholesterol biosynthesis is decreased in Cyp27a1^−/−^Cyp46a1^−/−^Soat1^−/−^ and Cyp27a1^−/−^Cyp46a1^−/−^Apoe^−/−^ mice, and represents a compensatory response to the triple gene ablations.

Next, we assessed the apolipoprotein-mediated retinal cholesterol traffic by measuring the mRNA for different apolipoproteins (Figure 5) and conducting retinal proteomics on Cyp27a1^−/−^Cyp46a1^−/−^Soat1^−/−^ vs. WT mice (Table 1; Table 2). Changes in the gene expression were sex-specific (Figure 5) and for apolipoproteins were not translated into changes in the protein expression as exemplified by APOAs, APOE, and APOJ, the major retinal apolipoproteins (Table 1). Rather, retinal proteomics pointed to enhanced vesicle formation in the Cyp27a1^−/−^Cyp46a1^−/−^Soat1^−/−^ vs. WT retina as an expression of 7 proteins from the endosomal, secretory, and protrusion formation pathways (CHGA, HDAC6, PAFAH1B, SEPT6, SRP19, TAGLN2, and TAGLN3, Figure 6) [41,42,43,44,45,46,47,48,49,50] was increased in the former (Table 2). Hence, retinal vesicle formation was investigated by TEM and found to be most prominent in the Cyp27a1^−/−^Cyp46a1^−/−^Soat1^−/−^ retina followed by Cyp27a1^−/−^Cyp46a1^−/−^Apoe^−/−^ and then WT retina (Figure 9; Figure 10). Notably, in the Cyp27a1^−/−^Cyp46a1^−/−^Soat1^−/−^ retina, vesicles were detected at both apical and basal RPE sides, possibly a reflection of the bi-directional traffic to the intraretinal and choroidal vascular networks. Traffic to the intraretinal vasculature was further supported by abundant vesicles in the intraretinal space of the outer nuclear layer and neurovascular units of the ganglion cell layer (Figure 9 and Figure 10). Also, the expression of HDAC6, which was found in the apically secreted extracellular vesicles derived from the oxidatively stressed RPE [51], was increased 3-fold (Table 2) in the Cyp27a1^−/−^Cyp46a1^−/−^Soat1^−/−^ retina.

Extracellular vesicles are generally enriched with cholesterol as compared to other cellular membranes, and their release is usually enhanced from cells enriched with cholesterol [52]. Therefore, recently, extracellular vesicles were suggested to be a part of cellular machinery ensuring cholesterol homeostasis [52]. Yet, in vivo support for cholesterol removal via extracellular vesicles is still scarce and requires more evidence. We blocked the major pathways of retinal cholesterol output (Figure 1) and created unique conditions, in which a compensatory downregulation of retinal cholesterol biosynthesis was probably not sufficient to normalize retinal cholesterol levels. Hence the formation of vesicles, possibly a reserve mechanism, was increased, and likely contributed to lowering of retinal cholesterol. If so, the present work appears to be the first in vivo study in which increased vesicle formation could be correlated with cholesterol lowering. We also identified an organ, the retina, where this mechanism could be operative. 

Vesicle formation requires energy [53], and remarkably, retinal proteomics identified 5 proteins (ACAA2, ASAH1, GAA, GALE, and PDHX, Figure 7) [54,55,56,57,58], which were upregulated in the Cyp27a1^−/−^Cyp46a1^−/−^Soat1^−/−^ vs. WT retina (Table 2) and participate in glucose production and utilization as well as fatty acid β-oxidation (Table S1). If energy homeostasis is indeed affected in the Cyp27a1^−/−^Cyp46a1^−/−^Soat1^−/−^ retina, there should be an increased production of ATP due to glycolysis and increased acetyl CoA availability as the latter is necessary for the tricarboxylic acid cycle, which provides NADPH for ATP production during mitochondrial (Figure 7) or extra mitochondrial oxidative phosphorylation [59,60]. Also, extracellular vesicles contain proteins [61], therefore enhanced vesicle formation will lead to retinal protein loss, if this loss is not compensated for, a possible reason for an increase in abundance of the overwhelming majority of proteins in the Cyp27a1^−/−^Cyp46a1^−/−^Soat1^−/−^ vs. WT retina (Table 1). The expression of LAMTOR3, PSAT, BPHL, and SLC14A1 [62,63,64,65], which are involved in protein homeostasis (Figure 7, Suppl. T1), was increased in the Cyp27a1^−/−^Cyp46a1^−/−^Soat1^−/−^ retina (Table 2) and could be the consequence of the enhanced extracellular vesicle formation. Thus not only the formation of extracellular vesicles seemed to be enhanced in the Cyp27a1^−/−^Cyp46a1^−/−^Soat1^−/−^ retina (Figure 6) but also the processes which are required (i.e., ATP production) and affected (i.e., protein homeostasis and ubiquitination) by vesicle formation.

**Figure 11:**
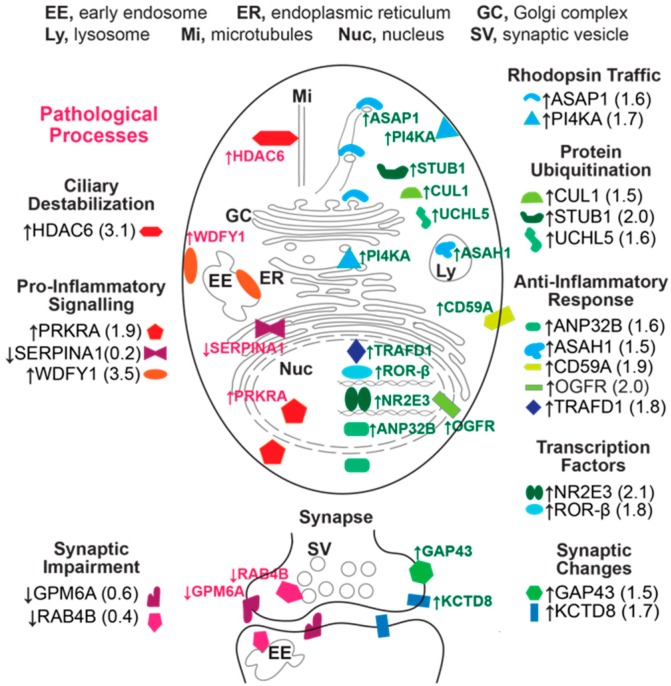
Differentially expressed proteins in the Cyp27a1^−/−^Cyp46a1^−/−^Soat1^−/−^ vs. WT retina of pertinence to retinal structure and function. Pathological processes that could be triggered by changes in protein abundance are on the left-hand side; all other processes are on the right-hand side. Protein full names and all the supporting references are provided in the Text S1. See Discussion for explanation. HDAC6 deacetylates α-tubulin, a main microtubule component, and thereby promote microtubule disassembly. PRKRA and WDFY participate in the activation of NF-κB, a transcription factor and key regulator of many important cellular processes including immune response, synaptic plasticity, memory, cytokine production, and other. SERPINA1 is a serine peptidase inhibitor, which can act as an anti-inflammatory agent. GPM6A and RAB4B are involved in synaptogenesis. ASAP1 plays a principal role in vesicular transport of rhodopsin and other rhodopsin-like receptors from the Golgi complex to cilia. PI4KA is an important enzyme for phototransduction and its downstream products can affect ASAP1. CUL1 is an E3 ubiquitin ligase, which can interact with STUB1 and protect cells from degeneration and apoptosis. STUB1 is another E3 ubiquitin-protein ligase which targets misfolded chaperone substrates towards proteasomal degradation promoting neuronal survival under conditions of oxygen and glucose deprivation. UCHL5 is a deubiquitinating enzyme that inhibits apoptosis and plays a role in maintenance of synaptic function. ANP32B is a multifunctional protein involved in immunomodulation, regulation of transcription, and regulation of apoptosis; it has pro-survival activity which correlates with its ability to inhibit caspase 3. ASAH1 is a lysosomal ceramidase, whose upregulation could rescue RPE from oxidative stress; its deficiency leads to retinal inflammation and severe visual impairment. CD59A, a membrane protein, is known to protect cells from membrane attack complex, hence suppressing inflammation and NF-κB activation; in the retina, CD59A can counteract retinal cell degeneration induced by ischemia reperfusion injury. OGFR is a receptor, which can regulate immune response and reduce astroglyosis, a sign in many cases of inflammation. TRAFD1 is an interferon- and LPS-inducible protein and a negative feedback regulator of NF-κB signaling, which limits excessive immune response. NR2E3 is a photoreceptor-specific nuclear receptor, which plays a critical role in retinogenesis and regulation of the genes involved in phototransduction in mature retina. RORβ is an orphan receptor expressed in the retina during embryogenesis and necessary for both cone and rod differentiation. GAP43 is a synaptic protein associated with increased synaptogenesis and axonal growth. KCTD8 is the auxiliary GABA(B) receptor subunit that can modulate receptor response and agonist-dependent desensitization. Altered abundance of all the proteins indicated in this figure can affect survival and function of retinal cells.

The present work provides insights into potential mechanisms that could underlie the deteriorations in retinal function in Cyp27a1^−/−^Cyp46a1^−/−^Soat1^−/−^ mice (Figure 3). Indeed, increased expression of HDAC6, a α-tubulin deacetylase, can perhaps lead to ciliary destabilization and potentially impair the traffic between the photoreceptor inner and outer segments [66,67]. In turn, these potential effects on the cilia can be compensated, fully or in part, by an increased expression of the proteins involved in rhodopsin traffic (ASAP1 and PI4KA), protein ubiquitination (CUL1, STUB1, and UCHL5) as well as the photoreceptor gene transcription (NR2E3 and RORβ) [68,69,70,71,72,73,74,75,76]. In addition, impaired ERG function could reflect a balance between the negative (decreased expression GPM6A and RAB4B) and positive (increased expression of GAP43 and KCTD8) effects on synaptic processes [77,78,79,80]. Inflammation may lead to the photoreceptor loss and thereby affect the ERG as well. The pro-inflammatory signaling is indicated by the upregulation of PRKRA and WDFY, which participate in the activation of NF-κB [81,82], a transcription factor and key regulator of immune and other functions, as well as a decreased abundance of SERPINA1E (an acute phase protein), which can attenuate microglia-mediated retinal degeneration [83]. The anti-inflammatory response could be due to the upregulation of ANP32B, ASAH1, CD59A, OGFR, and TRAFD1, proteins that play different roles (Figure 11, Table S1) but all affecting inflammation [84,85,86,87,88,89]. Thus, retinal proteomics also identified the non cholesterol-related proteins and processes that can be affected by blockage of the pathways of retinal cholesterol metabolism and storage. Further studies are required to target more precisely the major contributors to changes in the Cyp27a1^−/−^Cyp46a1^−/−^Soat1^−/−^ retina. In Cyp27a1^−/−^Cyp46a1^−/−^Apoe^−/−^ mice, retinal function was dependent on sex and was normal in female animals (Figure 3), in agreement with our previous findings that APOE absence elicits strong compensatory mechanisms that minimize retinal impact of this protein absence [27]. Future work will map these responses by retinal proteomics in Cyp27a1^−/−^Cyp46a1^−/−^Apoe^−/−^ mice for comparison to Apoe^−/−^ results [27].

**Figure 12:**
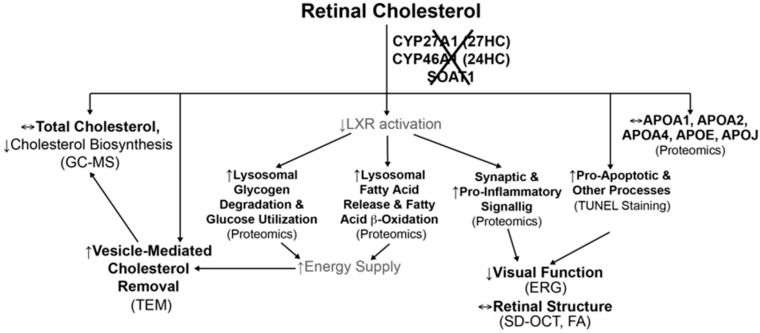
Proposed model unifying different processes in the Cyp27a1^−/−^Cyp46a1^−/−^Soat1^−/−^ retina. Processes with an experimental support (in parenthesis) are in black font and those that are putative are in gray font. See Discussion for explanation. 27HC and 24HC, 27- and 24-hydroxycholesterol, respectively. GC-MS, gas chromatography-mass spectrometry; ERG, electroretinography; TEM, transmission electron microscopy.

We propose the following model (Figure 12) to unify the available data and to identify the questions that remain unanswered to completely explain changes in the Cyp27a1^−/−^Cyp46a1^−/−^Soat1^−/−^ retina. The absence of SOAT1 can increase the availability for the retina of fatty acids, which are used as the enzyme substrates, whereas the absence of CYP27A1 and CYP46A1 abolishes the production of 27-hydroxycholesterol and 24-hydroxycholesterol, which are activating ligands for LXRs. LXRs are important transcription factors that play essential roles in the regulation of cholesterol metabolism and also integration of glucose and fatty acid metabolism as well as immune and inflammatory responses [90]. Accordingly, glucose utilization and fatty acid β-oxidation could be increased in the Cyp27a1^−/−^Cyp46a1^−/−^Soat1^−/−^ retina as a compensatory response to decreased LXR activation and/or increased fatty acid availability. This will lead to an increased energy production, which could be used to form extracellular vesicles, an energy-consuming process and alternate means to eliminate excess cellular cholesterol when the compensatory responses from cholesterol biosynthesis and efflux to LP are either not sufficient or not operative. In addition, decreased LXR activation could promote proinflammatory processes and affect retinal function. Apparently, other processes could also become affected in the Cyp27a1^−/−^Cyp46a1^−/−^Soat1^−/−^ retina as suggested by retinal proteomics (Figure 11). Future studies are needed to address these outstanding questions.

## 5. Conclusions

We comprehensively characterized the retina of Cyp27a1^−/−^Cyp46a1^−/−^Soat1^−/−^ and Cyp27a1^−/−^Cyp46a1^−/−^Apoe^−/−^ mice and identified some of the homeostatic responses to the blockage of the major pathways of retinal cholesterol output and storage. The major cholesterol-related response included the upregulation of the pathways producing intracellular and extracellular vesicles, which seems to be a reserve mechanism for retinal cholesterol removal. The non-cholesterol-related responses pertained to retinal function, photoreceptor viability, and possibly retinal glucose as well as fatty acid metabolism. The data obtained provide insight into the links between retinal cholesterol homeostasis and other important retinal processes. Notably, the content of total retinal cholesterol does not seem to predict retinal abnormalities, and it is rather the network of compensatory mechanisms that appears to determine the retinal phenotype.

## Figures and Tables

**Table 1:** Proteins detected by the label-free approach that were analyzed for gene expression in the Cyp27a1^−/−^Cyp46a1^−/−^Soat1^−/−^ (TKO) and C57BL/6J (WT) mouse retinas.

Protein	№ of Identified Peptides	Sequence Coverage (%)	Peptide Intensity (a.u.) × 10^6^		TKO/WT, Protein Ratio	*p*
			TKO	WT		
APOA 1	7	36	3.55 ± 1.63	3.63 ± 0.69	0.98	0.94
APOA 2	1	10	0.95 ± 0.55	0.32 ± 0.16	2.99	0.13
APOA4	1	3	0.09 ± 0.15	0.18 ±0.17	0.47	0.49
APOE	7	24	1.52 ± 0.21	1.56 ± 0.26	0.98	0.86
APOJ	7	20	3.17 ± 0.11	2.66 ± 0.19	1.12	0.56

**Table 2:** Proteins with significant changes in abundance (≥1.5-fold) in the Cyp27a1^−/−^Cyp46a1^−/−^Soat1^−/−^ (TKO) vs. C57BL/6J (WT) retina. Protein grouping is by process and shows each protein only in one group despite the involvement in multiple processes. Proteins with a decreased expression are in bold.

Protein	№ of Peptides With Significant Changes in Abundance	№ of Unique Peptides	Sequence Coverage (%)	Peptide Intensity (a.u.) × 10^6^		TKO/WT, Protein Ratio
				TKO	WT	
	**Cytoskeletal organization, vesicular and secretory pathway**					
ASAP1	2	2	3	1.0 ± 0.1	0.6 ± 0.1	1.6
ATP1A2	28	12	35	4.9 ± 1.7	1.6 ± 0.5	3.1
CHGA	2	2	9	2.6 ± 0.1	1.6 ± 0.5	1.6
HDAC6	3	3	5	0.9 ± 0.2	0.3 ± 0.2	3.1
PAFAH1B2	5	5	42	13.3 ± 1.7	8.8 ± 1.2	1.5
PI4KA	10	10	6	1.5 ± 0.2	0.9 ± 0.1	1.7
SEPT6	11	6	37	6.5 ± 0.2	4.1 ± 0.8	1.6
SRP19	3	3	35	1.2 ± 0.2	0.6 ± 0.2	2.1
TAGLN2	9	6	54	2.5 ± 0.4	0.9 ± 0.2	2.8
TAGLN3	12	9	71	8.2 ± 0.7	5.5 ± 1.0	1.5
TINAGL1	3	3	12	0.7 ± 0.1	0.3 ± 0.1	2.2
**Energy Homeostasis**						
ACAA2	15	15	59	8.8 ± 1.4	5.9 ± 0.7	1.5
ASAH1	7	7	22	2.1 ± 0.3	1.4 ± 0.2	1.5
BPHL	3	3	19	1.1 ± 0.2	0.5 ± 0.1	2.1
GAA	3	3	6	1.0 ± 0.2	0.7 ± 0.1	1.5
GALE	3	3	15	1.1 ± 0.1	0.6 ± 0.1	1.9
LAMTOR3	2	2	31	1.1 ± 0.1	0.4 ± 0.1	2.8
PDHX	6	6	17	3.2 ± 0.5	1.8 ± 0.1	1.8
PSAT1	18	18	59	9.1 ± 1.8	4.8 ± 1.1	1.9
SLC14A1	4	4	9	2.0 ± 0.5	1.1 ± 0.2	1.8
**DNA/RNA Processing, Transcription, Regulation of Transcription and Translation**						
EEF1D	9	8	47	7.6 ± 1.6	4.8 ± 0.5	1.6
GTF2E1	3	3	9	0.5 ± 0.0	0.3 ± 0.1	1.7
NIPBL	2	2	1	0.4 ± 0.0	0.1 ± 0.2	3.2
NR2E3	6	6	26	1.9 ± 0.5	0.9±0.3	2.1
NXF1	3	3	9	1.1 ± 0.1	0.6 ± 0.2	1.6
PDS5A	3	3	5	0.4 ± 0.1	0.2 ± 0.0	2.0
RORB	4	4	11	0.9 ± 0.2	0.5 ± 0.1	1.8
RPA3	2	2	33	0.5 ± 0.1	0.1 ± 0.2	3.7
U2AF2	10	10	36	16.2 ± 0.3	10.2 ± 2.2	1.6
**Inflammation**						
ANP32B	6	5	24	10.7 ± 0.8	6.9 ± 1.2	1.6
CD59A	2	2	21	3.0 ± 0.6	1.6 ± 0.4	1.9
OGFR	3	3	7	0.5 ± 0.0	0.3 ± 0.1	2.0
PRKRA	3	3	15	1.5 ± 0.1	0.8 ± 0.3	1.9
**SERPINA1E**	**10**	**4**	**35**	**0.7 ± 0.7**	**3.6 ± 1.4**	**0.2**
TRAFD1	3	3	8	1.5 ± 0.2	0.9 ± 0.2	1.8
WDFY1	4	4	12	1.4 ± 0.4	0.4 ± 0.1	3.5
**Synaptic function**						
GAP43	10	10	48	3.3 ± 0.1	2.2 ± 0.2	1.5
**GPM6A**	**7**	**7**	**23**	**28.8 ± 6.9**	**50.9 ± 6.2**	**0.6**
KCTD8	2	2	8	0.7 ± 0.1	0.4 ± 0.1	1.7
**RAB4B**	**4**	**2**	**25**	**0.5 ± 0.2**	**1.4 ± 0.3**	**0.4**
**Ubiquitination**						
CUL1	9	9	18	1.2 ± 0.1	0.8 ± 0.2	1.5
STUB1	6	6	25	1.3 ± 0.3	0.6 ± 0.2	2.0
UCHL5	5	5	28	1.9 ± 0.3	1.2 ± 0.3	1.6

## Supplementary Materials

The following are available online at https://www.mdpi.com/2218-273X/9/12/867/s1. Supporting Text S1: Gene abbreviations, protein abbreviations, and functions of proteins indicated in the present work; Supplemental Table S1: List of primers for quantitative real-time PCR; Supplemental Table S2: Statistical analyses by two-way ANOVA with Bonferroni correction of the serum total cholesterol content in different genotypes; Supplemental Table S3: Statistical analyses by two-way ANOVA with Bonferroni correction of the retinal total cholesterol content in different genotypes; Supplemental Table S4: Statistical analyses by two-way ANOVA with Bonferroni correction of the retinal free lathosterol content in different genotypes; Supplemental Table S5. Statistical analyses by two-way ANOVA with Bonferroni correction of the retinal free desmosterol content in different genotypes.
