# Supplementary material for: The Interplay between Retinal Pathways of Cholesterol Output and Its Effects on Mouse Retina
Source: Biomolecules. 2019 Dec 12;9(12):867. doi: 10.3390/biom9120867 (PMC6995521; doi:10.3390/biom9120867)
Supplement: Supplementary file 1 [file biomolecules-09-00867-s001.pdf]

Supplemental Information

The interplay between retinal pathways of cholesterol output and its effects on mouse retina

Alexey M. Petrov, Artem A. Astafev, Natalia Mast, Aicha Saadane, Nicole El-Darzi, and Irina A. Pikuleva

Department of Ophthalmology and Visual Sciences, Case Western Reserve University, Cleveland, OH 44106, USA

Supporting Text T1. Gene abbreviations, protein abbreviations, and functions of proteins indicated in the present work. Gene symbols are italicized and begin with an uppercase letter; protein symbols have all letters in uppercase.

Gene abbreviations

Abca1 (ATP binding cassette subfamily A member 1); *Apoa1*, *Apoa2*, *Apoa4*, *Apob*, *Apoc3*, *Apod*, *Apoe*, *ApoJ* (apolipoproteins A1, A2, A4, B, C3, D, E and J, respectively); *Ccl2* (C-C motif chemokine 2); *Cox-2* (prostaglandin G/H synthase 2); *Hmgcr* (3-hydroxy-3-methylglutaryl-CoA reductase); *Il-6* (interleukin-6)*; Srebp2* (sterol regulatory element-binding protein 2)*; Tnfα* (tumor necrosis factor *α*).

Protein abbreviations

ACAA2 (3-ketoacyl-CoA thiolase); ANK2 (ankyrin B); ANP32B (acidic leucine-rich nuclear phosphoprotein 32 family member B); APOA1, APOA2, APOA4, APOE (apolipoproteins A1, A2, A4 and E, respectively); ASAH1 (acid ceramidase); ASAP1 (arf-GAP with SH3 domain ANK repeat and PH domain-containing protein 1); ATP1A2 (sodium/potassium-transporting ATPase subunit alpha-2); BPHL (valacyclovir hydrolase); C1ORF123 (UPF0587 protein chromosome 1 open reading frame 23); CD59A (CD59A glycoprotein); CHGA (chromogranin-A), CUL1 (cullin-1); CYP27A1 (cytochrome P450 family 27 subfamily A member 1); CYP46A1 (cytochrome P450 family 46 subfamily A member 1); E2A (transcription factor 3); EEF1D (elongation factor 1-delta); EF-1α (elongation factor 1-alpha); EGFR (epidermal growth factor receptor); EZR (ezrin); GAA (lysosomal alpha-glucosidase); GALE (UDP-galactose 4-epimerase); GAP43 (growth associated protein 43 or neuromodulin); GPM6A (neuronal membrane glycoprotein M6-a); GTF2E1 (general transcription factor IIE subunit 1); HDAC6 (histone deacetylase 6); KCTD8 (BTB/POZ domain-containing protein KCTD8); LAMTOR3 (ragulator complex protein LAMTOR3); LXR (liver X receptor); mTORC1 (mammalian target of rapamycin complex 1); NF-kB (Nuclear Factor Kappa B Subunit 1); NIPBL (nipped-B-like protein; cohesin loading factor); NR2E3 (photoreceptor-specific nuclear receptor); NRL (neural retina leucine zipper); NXF1 (nuclear RNA export factor 1); OGF (opioid growth factor); OGFR (opioid growth factor receptor); PAFAH1B2 (platelet-activating factor acetylhydrolase IB subunit beta); PDHX (pyruvate dehydrogenase protein X component); PDS5A (sister chromatid cohesion protein PDS5 homolog A); PI4KA (phosphatidylinositol 4-kinase alpha); PKR (protein kinase R); PLC (phospholipase C); PRKRA (interferon-inducible double-stranded RNA-dependent protein kinase activator A); PSAT1 (phosphoserine aminotransferase); RAB4B (ras-related protein Rab-4B2); RHOA (ras homolog gene family; member A); RORβ (nuclear receptor ROR β); RPA3 (replication protein A 14 kDa subunit); SEPT6 (septin-6); SERPINA1E (α-1-antitrypsin 1-5); SKP1 (S-phase kinase-associated protein 1); SLC14A1 (urea transporter 1); SOAT1 (sterol-O-acyltransferase or acyl-coenzyme A: cholesterol acyltransferase); SRP19 (signal recognition particle 19 kDa); STUB1 (STIP1 homology and U box-containing protein 1 or CHIP); TAGLN2 (transgelin-2); TAGLN3 (transgelin-3); TINAGL1 (tubulointerstitial nephritis antigen-like); TLR (toll-like receptor); TRAFD1 (tRAF-type zinc finger domain-containing protein 1); U2AF2 (splicing factor U2AF 65 kDa subunit); UCHL5 (ubiquitin carboxyl-terminal hydrolase isozyme L5); WDFY1 (WD repeat and FYVE domain-containing protein 1).

Protein functions

Cytoskeletal organization, vesicular, and secretory pathway

ASAP1, a phosphatidylinositol 4,5-bisphosphate-dependent Arf GTPase-activating protein involved in vesicular transport of rhodopsin from the Golgi complex to cilia in photoreceptor cells [1,2].

ATP1A2, the catalytic α2 subunit of the Na+/K+ pump responsible for establishing and maintaining the electrochemical gradients of Na+ and K+ ions across the plasma membrane. In the photoreceptors, Na+/K+ pump interacts with membrane cytoskeleton via ankyrin that tethers the pump to spectrin and actin cytoskeleton [3].

HDAC6, a tubulin-specific deacetylase, which is a critical component for the cilia disassembly *via* tubulin deacetylation. HDAC6 also deacetylases actin-regulatory protein cortactin, thereby stimulating actin polymerization. The latter contributes to cilia disassembly and membrane ruffle (protrusion) formation [4-6]. HDAC6 was found in extracellular vesicles derived from oxidatively stressed RPE [7]. HDAC6 inhibition can protect retinal cells from oxidative stress as well as from ischemia and reperfusion injury [8,9].

CHGA, a neuroendocrine secretory protein, which is required for secretory granule formation and positively controls a number of formed granules [10].

PAFAH1B2, a catalytic subunit 2 of Platelet Activating Factor Acetylhydrolase (PAFAH) Ib having the phospholipase A2 activity. Activity of this complex is required for endosome membrane tube formation and integrity of Golgi complex, thereby contributing to cargo recycling *via* endosomal route and maintaining secretion, respectively [11-13].

PI4KA, a phosphatidylinositol (PI) 4-kinase, which catalyzes the first step in the biosynthesis of phosphatidylinositol 4,5-bisphosphate. It is mostly membrane-bound and located at the endoplasmic reticulum. PK4KA is involved in membrane traffic and phototransduction [14-17].

SEPT6, a member of the filament-forming GTP-binding proteins implicated in cytoskeleton and membrane organization; SEPT6 binds to F-actin and is required for multivesicular body maturation from early endosomes. SEPT6 also triggers filopodia formation by increasing the recruitment of cortactin, a regulator of actin polymerization [18,19].

SRP19, an essential protein for the assembly of the signal recognition particle, a conserved ribonucleoprotein complex that mediates the translation and targeting of secretory and membrane proteins to endoplasmic reticulum for the further incorporation into the secretory pathway [20,21].

TAGLN2 and TAGLN3, members of the actin-binding protein family. TAGLN2 and TAGLN3 stimulate G-actin polymerization that can enhance formation of the filopodia-like membrane protrusion and processes [22-25].

TINAGL1, a secreted extracellular protein and a ligand for integrins; regulates cell adhesion [26,27].

Energy homeostasis

ACAA2, a mitochondrial enzyme, which catalyzes the last step of the mitochondrial fatty acid β-oxidation. The oxidation of fatty acids in the brain is limited by the activity of the ACAA2 [28].

ASAH1, a lysosomal ceramidase that hydrolyzes ceramides into sphingosine and free fatty acids [29]. Deficiency in ASAH1 leads to retinal inflammation and severe visual impairment [30]. Induction of ASAH1 could rescue retinal pigment epithelium from oxidative stress by hydrolyzing ceramide excess, which is accumulated in response to oxidative stress and could induce cell death [31].

BPHL, a mitochondrial serine hydrolase, which can hydrolyze a highly toxic homocysteine thiolactone, a byproduct of protein biosynthesis, to homocysteine [32,33].

GAA, a lysosomal α-glucosidase, which cleaves glycogen to glucose in lysosomes. Deficiency of GAA leads to Pompe disease, characterized by accumulation of glycogen in the lysosomes [34].

GALE, a cytosolic enzyme, which catalyzes the reversible epimerization of UDP-galactose and UDP-glucose. GALE plays a critical role in the Leloir pathway of galactose catabolism, in which galactose is converted to glucose 1-phosphate, a precursor of glucose 6-phosphate [35].

LAMTOR3, a part of the Ragulator complex, which along with V-ATPase (a proton pump) is involved in amino acid sensing in the lysosomes [36].

PDHX, a component X of the pyruvate dehydrogenase complex, which catalyzes the irreversible oxidation of pyruvate to acetyl CoA. This reaction is rate-limiting under aerobic conditions for the oxidative removal of glucose and pyruvate [37].

PSAT1, a member of the class-V pyridoxal-phosphate-dependent aminotransferase family. PSAT1 catalyzes the reversible conversion of 3-phosphohydroxypyruvate to phosphoserine, which is important for serine biosynthesis [38].

SLC14A1, a membrane urea transporter, whose elevated levels can represent a response to the elevated urea content inside cells [39,40].

Transcription factors

NR2E3, a photoreceptor-specific nuclear receptor, which plays a key role in photoreceptor development and regulates the expression of genes involved in phototransduction in mature retina [41,42].

RORβ, an orphan nuclear receptor β, which is expressed in throughout the retina during embryogenesis with a peak expression at neonatal stages. RORβ may play a role in the differentiation of many retinal cells; its role in cone and rod differentiation is documented [43,44].

Inflammation

ANP32B, a multifunctional protein, is involved in immunomodulation, regulation of transcription and apoptosis. ANP32B has pro-survival activity which correlates with its ability to inhibit caspase 3 [45-47].

CD59A, a potent membrane-bound inhibitor of the complement membrane attack complex. This complex can activate NF-κB signaling and increases inflammation [48]. Deficiency in CD59A can increase accumulation of subretinal macrophages and/or microglia during aging [49]. CD59A can protect retinal cells from degeneration induced by ischemia reperfusion injury and could also counteract laser-induced choroidal neovascularization [50-52].

OGFR, the opioid growth factor receptor localized to the outer nuclear membrane. This receptor plays an immunomodulatory role, and its activation can inhibit astrocyte proliferation and astrogliosis, which can enhance inflammation [53,54].

PRKRA, an interferon-inducible double-stranded RNA-dependent protein kinase activator A. Activation of this kinase can stimulate NF-κB-dependent pathways leading to induction of pro-inflammatory cytokines [55].

SERPINA1E, a serine peptidase inhibitor, which has anti-inflammatory properties [56]. SERPINA1E can attenuate microglia-mediated neuroinflammation and retinal degeneration in Rd1 mice, a mouse model of retinitis pigmentosa [57]. Also, SERPINA1E reduced inflammation and delayed ganglion cell loss and retinal thinning in a mouse model of diabetic retinopathy [58].

TRAFD1, an interferon and LPS-inducible gene, is a negative feedback regulator of the toll receptor (TLR) and NF-kB signaling. TRAFD1 can limit immune response [59,60].

WDFY1, a crucial adaptor protein in the TLR3/4 signaling pathway. Overexpression of WDFY1 potentiates TLR3- and TLR4-mediated activation of NF-κB, interferon regulatory factor 3, and production of type I interferons as well as inflammatory cytokines [61,62].

Synaptic function

GAP43, a synaptic protein and indicator of neurite elongation (axonal growth) and synapse formation. GAP43 is highly expressed during embryogenesis [63] and is upregulated during axonal regeneration, suggesting a role in regenerative responses [64].

GPM6A, a neuronal transmembrane protein which promotes various cellular protrusion formation such as neurites, filopodia and dendrite spines in an actin-independent manner [65,66]. GPM6A resides in lipid rafts and induces the clustering of lipid rafts [67]. The association of GPM6A with lipid rafts is important for its role in filopodia formation [68]. GPM6A is required for synaptic spine formation, axonal outgrowth, and synaptogenesis [69].

KCTD8, an auxiliary GABA(B) receptor subunit that modulates the receptor response, basal activity of the receptor, and its agonist-dependent desensitization [70,71].

RAB4B, a small GTPase that participates in sorting of cargo from early to recycling endosomes; the membrane material is then recycled back to the plasma membrane [72,73]. Also, RAB4B is required for synaptic spine

maintenance, and decreased expression of RAB4B correlates with reduced dendrite branching in neurons [74,75]. RAB4 can also be involved in axonal growth [76].

Ubiquitin proteasome system and protein folding

CUL1, a core component of multiple cullin-RING-based SCF (SKP1-CUL1-F-box protein) E3 ubiquitin-protein ligase complexes. These complexes can protect cells from apoptosis and neurodegeneration [77,78]. CUL1 can directly interact with STUB1 in the E3 ligase complex [79]

STUB1, an E3 ubiquitin-protein ligase CHIP, which targets misfolded chaperone substrates towards proteasomal degradation. STUB1 can protect neurons from neurodegeneration induced by misfolded proteins, mitochondrial dysfunction induced by oxygen and glucose deprivation, and death induced by oxidative stress [80-83].

UCHL5, a deubiquitinating enzyme. Inhibition of UCHL5 can induce proteotoxic stress and apoptosis [84-86]. It associates with synaptic proteasomes and participates in synaptic plasticity [87,88].

Supplemental Table S1. List of primers for quantitative real-time PCR.

Gene	Forward primer	Reverse primer
Abca1	5'-CAGGAAGCACGTGTCTGAAG-3'	5'-GTGGTCTCCGAGATGCCATA-3'
Apoa1	5'-GGGTTCAACCGTTAGTCAGC-3'	5'-TGGAATTCGTCCAGGTAGGG-3'
Apoa2	5'-CTGTAGCCTGGAAGGAGCTT-3'	5'-AGGTCTTGGCCTTCTCCATC-3'
Apoa4	5'-GAGCCCATGGGAGAGATGTT-3'	5'-TCTCCAGGAAGCTCAAGTGG-3'
Apob	5'-ACCAACCAGATCGTGGGAAT-3'	5'-GCCATCTTGCAGGTCAGAAG-3'
Apoc3	5'-CACCGGCTTCTGGGATTCTA-3'	5'-GAACAGGCACATCTGCAACA-3'
Apod	5'-TGAAGCCAAACAGAGCAACGT-3'	5'-GGCATCAACGGGAAGAACTG-3'
Apoe	5'-AACAGACCCAGCAAATACGC-3'	5'-TGTTGTTGCAGGACAGGAGA-3'
Apoj	5'-ATGATCCACCAGGCTCAACA-3'	5'-GTGCGGTCATCTTCACCTTC-3'
β-Actin	5'-TGTTACCAACTGGGACGACATG-3'	5'-TTGTAGAAGGTGTGGTGCCAGA-3'
Ccl2	5'-TCACTGAAGCCAGCTCTCTCTTC-3'	5'-GTGAACAGCAGGCCCAGAA-3'
Cox-2	5'-TCCTCCACTCATGAGCAGTC-3'	5'-AACCCTGGTCGGTTTGATGT-3'
Hmgcr	5'-TTGGTCCTTGTTCACGCTCAT-3'	5'-TTCGTCCAGACCCAAGGAAAC-3'
Il-6	5'-AGTGGCTAAGGACCAAGACC-3'	5'-ACCACAGTGAGGAATGTCCA-3'
Srebp2	5'-CAGCTGGATCCTCCCAAAGA-3'	5'-CTCAGAACGCCAGACTTGTG-3'
Tnfα	5'-CTCATGCACCACCATCAAGG-3'	5'-ACCTGACCACTCTCCCTTTG-3'

Supplemental Table S2. Statistical analyses by two-way ANOVA with Bonferroni correction of the serum total cholesterol content in different genotypes. F, female; M, male; MB, mixed (C57BL/6J;129S6/SvEv) background; N/A, non applicable; ns, non significant; WT wild type. *$P \leq 0.05$, **$P \leq 0.01$, ***$P \leq 0.001$

Genotype	C57BL/6J, F	C57BL/6J, M	*Soat1*$^{-/-}$, F	*Soat1*$^{-/-}$, M	*ApoE*$^{-/-}$, F	*ApoE*$^{-/-}$, M	MB WT, F	MB WT, M	*Cyp27a1*$^{-/-}$ *Cyp46a1*$^{-/-}$, F	*Cyp27a1*$^{-/-}$ *Cyp46a1*$^{-/-}$, M	*Cyp27a1*$^{-/-}$ *Cyp46a1*$^{-/-}$ *Soat1*$^{-/-}$, F	*Cyp27a1*$^{-/-}$ *Cyp46a1*$^{-/-}$ *Soat1*$^{-/-}$, M	*Cyp27a1*$^{-/-}$ *Cyp46a1*$^{-/-}$ *ApoE*$^{-/-}$, F	*Cyp27a1*$^{-/-}$ *Cyp46a1*$^{-/-}$ *ApoE*$^{-/-}$, M
C57BL/6J, F	N/A	ns	ns	*	***	***	ns	ns	ns	ns	ns	***	***	***
C57BL/6J, M	ns	N/A	ns	ns	ns	***	ns	ns	ns	ns	ns	ns	ns	***
Soat1$^{-/-}$, F	ns	ns	N/A	ns	***	***	ns	ns	ns	ns	ns	ns	***	***
Soat1$^{-/-}$, M	*	ns	ns	N/A	ns	***	ns	ns	ns	ns	ns	ns	ns	***
ApoE$^{-/-}$, F	***	***	***	***	N/A	***	***	***	ns	***	ns	**	ns	***
ApoE$^{-/-}$, M	***	***	***	***	***	N/A	ns	***	ns	ns	ns	ns	ns	ns
MB WT, F	ns	ns	ns	*	***	***	N/A	ns	ns	ns	ns	**	ns	***
MB WT, M	ns	ns	ns	ns	***	***	ns	N/A	ns	ns	ns	ns	ns	ns
Cyp27a1$^{-/-}$ *Cyp46a1*$^{-/-}$, F	ns	ns	ns	ns	***	***	ns	ns	N/A	ns	ns	*	***	***
Cyp27a1$^{-/-}$ *Cyp46a1*$^{-/-}$, M	ns	ns	ns	ns	***	***	ns	ns	ns	N/A	ns	ns	ns	***
Cyp27a1$^{-/-}$ *Cyp46a1*$^{-/-}$ *Soat1*$^{-/-}$, F	ns	ns	ns	ns	***	***	ns	ns	ns	ns	N/A	ns	***	***
Cyp27a1$^{-/-}$ *Cyp46a1*$^{-/-}$ *Soat1*$^{-/-}$, M	***	ns	ns	ns	**	***	**	ns	*	ns	ns	N/A	ns	***
Cyp27a1$^{-/-}$ *Cyp46a1*$^{-/-}$ *ApoE*$^{-/-}$, F	***	***	***	***	ns	***	***	***	***	***	***	**	N/A	***
Cyp27a1$^{-/-}$ *Cyp46a1*$^{-/-}$ *ApoE*$^{-/-}$, M	***	***	***	***	***	ns	***	***	***	***	***	***	***	N/A

Supplemental Table S3. Statistical analyses by two-way ANOVA with Bonferroni correction of the retinal total cholesterol content in different genotypes. F, female; M, male; MB, mixed (C57BL/6J;129S6/SvEv) background; N/A, non applicable; ns, non significant; WT wild type. *$P \leq 0.05$, **$P \leq 0.01$, ***$P \leq 0.001$.

Genotype	C57BL/6J, F	C57BL/6J, M	*Soat1*$^{-/-}$, F	*Soat1*$^{-/-}$, M	*ApoE*$^{-/-}$, F	*ApoE*$^{-/-}$, M	MB WT, F	MB WT, M	*Cyp27a1*$^{-/-}$ *Cyp46a1*$^{-/-}$, F	*Cyp27a1*$^{-/-}$ *Cyp46a1*$^{-/-}$, M	*Cyp27a1*$^{-/-}$ *Cyp46a1*$^{-/-}$ *Soat1*$^{-/-}$, F	*Cyp27a1*$^{-/-}$ *Cyp46a1*$^{-/-}$ *Soat1*$^{-/-}$, M	*Cyp27a1*$^{-/-}$ *Cyp46a1*$^{-/-}$ *ApoE*$^{-/-}$, F	*Cyp27a1*$^{-/-}$ *Cyp46a1*$^{-/-}$ *ApoE*$^{-/-}$, M
C57BL/6J, F	N/A	ns	ns	ns	***	***	ns	ns	**	***	ns	ns	***	***
C57BL/6J, M	ns	N/A	ns	ns	***	***	ns	ns	**	***	ns	ns	***	***
Soat1$^{-/-}$, F	ns	ns	N/A	ns	***	***	ns	ns	***	***	ns	ns	***	***
Soat1$^{-/-}$, M	ns	ns	ns	N/A	***	***	ns	ns	***	***	ns	ns	***	***
ApoE$^{-/-}$, F	***	***	***	***	N/A	ns	***	***	***	***	***	***	***	ns
ApoE$^{-/-}$, M	***	***	***	***	ns	N/A	***	***	***	***	***	***	***	ns
MB WT, F	ns	ns	ns	ns	***	***	N/A	ns	***	***	ns	ns	***	***
MB WT, M	ns	ns	ns	ns	***	***	ns	N/A	***	***	ns	ns	***	***
Cyp27a1$^{-/-}$ *Cyp46a1*$^{-/-}$, F	**	**	***	***	***	***	***	***	N/A	ns	***	**	***	***
Cyp27a1$^{-/-}$ *Cyp46a1*$^{-/-}$, M	***	***	***	***	***	***	***	***	ns	N/A	***	***	ns	***
Cyp27a1$^{-/-}$ *Cyp46a1*$^{-/-}$ *Soat1*$^{-/-}$, F	ns	ns	ns	ns	***	***	ns	ns	***	***	N/A	ns	***	***
Cyp27a1$^{-/-}$ *Cyp46a1*$^{-/-}$ *Soat1*$^{-/-}$, M	ns	ns	ns	ns	***	***	ns	ns	**	***	ns	N/A	***	***
Cyp27a1$^{-/-}$ *Cyp46a1*$^{-/-}$ *ApoE*$^{-/-}$, F	***	***	***	***	***	***	***	***	***	ns	***	***	N/A	ns
Cyp27a1$^{-/-}$ *Cyp46a1*$^{-/-}$ *ApoE*$^{-/-}$, M	***	***	***	***	ns	ns	***	***	***	***	***	***	ns	N/A

Supplemental Table S4. Statistical analyses by two-way ANOVA with Bonferroni correction of the retinal free lathosterol content in different genotypes. F, female; M, male; MB, mixed (C57BL/6J;129S6/SvEv) background; N/A, non applicable; ns, non significant; WT wild type. *$P \leq 0.05$, **$P \leq 0.01$, ***$P \leq 0.001$.

Genotype	C57BL/6J, F	C57BL/6J, M	*Soat1*$^{-/-}$, F	*Soat1*$^{-/-}$, M	*ApoE*$^{-/-}$, F	*ApoE*$^{-/-}$, M	MIX WT, F	MIX WT, M	*Cyp27a1*$^{-/-}$ *Cyp46a1*$^{-/-}$, F	*Cyp27a1*$^{-/-}$ *Cyp46a1*$^{-/-}$, M	*Cyp27a1*$^{-/-}$ *Cyp46a1*$^{-/-}$ *Soat1*$^{-/-}$, F	*Cyp27a1*$^{-/-}$ *Cyp46a1*$^{-/-}$ *Soat1*$^{-/-}$, M	*Cyp27a1*$^{-/-}$ *Cyp46a1*$^{-/-}$ *ApoE*$^{-/-}$, F	*Cyp27a1*$^{-/-}$ *Cyp46a1*$^{-/-}$ *ApoE*$^{-/-}$, M
C57BL/6J, F	N/A	ns	ns	ns	***	***	ns	ns	ns	ns	***	***	***	***
C57BL/6J, M	ns	N/A	ns	ns	***	***	ns	ns	ns	ns	***	***	***	***
Soat1$^{-/-}$, F	ns	ns	N/A	ns	***	***	ns	ns	ns	ns	**	*	***	***
Soat1$^{-/-}$, M	ns	***	ns	N/A	***	***	ns	ns	ns	ns	**	*	***	***
ApoE$^{-/-}$, F	***	***	***	***	N/A	ns	***	***	***	***	***	***	***	***
ApoE$^{-/-}$, M	***	ns	***	***	ns	N/A	***	***	***	***	***	***	***	***
MIX WT, F	ns	ns	ns	ns	***	***	N/A	ns	ns	ns	***	*	***	***
MIX WT, M	ns	ns	ns	ns	***	***	ns	N/A	ns	ns	***	*	***	***
Cyp27a1$^{-/-}$ *Cyp46a1*$^{-/-}$, F	ns	ns	ns	ns	***	***	ns	ns	N/A	ns	***	***	***	***
Cyp27a1$^{-/-}$ *Cyp46a1*$^{-/-}$, M	ns	ns	ns	ns	***	***	ns	ns	ns	N/A	**	ns	***	***
Cyp27a1$^{-/-}$ *Cyp46a1*$^{-/-}$ *Soat1*$^{-/-}$, F	***	***	**	**	***	***	***	***	***	**	N/A	ns	***	***
Cyp27a1$^{-/-}$ *Cyp46a1*$^{-/-}$ *Soat1*$^{-/-}$, M	***	***	*	*	***	***	*	*	***	ns	ns	N/A	***	***
Cyp27a1$^{-/-}$ *Cyp46a1*$^{-/-}$ *ApoE*$^{-/-}$, F	***	***	***	***	***	***	***	***	***	***	***	***	N/A	ns
Cyp27a1$^{-/-}$ *Cyp46a1*$^{-/-}$ *ApoE*$^{-/-}$, M	***	***	***	***	***	***	***	***	***	***	***	***	ns	N/A

Supplemental Table S5. Statistical analyses by two-way ANOVA with Bonferroni correction of the retinal free desmosterol content in different genotypes. F, female; M, male; MB, mixed (C57BL/6J;129S6/SvEv) background; N/A, non applicable; ns, non significant; WT wild type. *$P \leq 0.05$, **$P \leq 0.01$, ***$P \leq 0.001$.

Genotype	C57BL/6J, F	C57BL/6J, M	*Soat1*$^{-/-}$, F	*Soat1*$^{-/-}$, M	*ApoE*$^{-/-}$, F	*ApoE*$^{-/-}$, M	MIX WT, F	MIX WT, M	*Cyp27a1*$^{-/-}$ *Cyp46a1*$^{-/-}$, F	*Cyp27a1*$^{-/-}$ *Cyp46a1*$^{-/-}$, M	*Cyp27a1*$^{-/-}$ *Cyp46a1*$^{-/-}$ *Soat1*$^{-/-}$, F	*Cyp27a1*$^{-/-}$ *Cyp46a1*$^{-/-}$ *Soat1*$^{-/-}$, M	*Cyp27a1*$^{-/-}$ *Cyp46a1*$^{-/-}$ *ApoE*$^{-/-}$, F	*Cyp27a1*$^{-/-}$ *Cyp46a1*$^{-/-}$ [illegible]
C57BL/6J, F	N/A	ns	ns	ns	***	***	ns	ns	ns	**	ns	ns	**	***
C57BL/6J, M	ns	N/A	ns	ns	***	***	ns	ns	ns	ns	ns	ns	***	***
Soat1$^{-/-}$, F	ns	ns	N/A	ns	***	***	ns	ns	ns	**	ns	ns	**	***
Soat1$^{-/-}$, M	ns	ns	ns	N/A	***	***	ns	ns	ns	*	ns	ns	**	***
ApoE$^{-/-}$, F	***	***	***	***	N/A	**	***	***	***	***	***	***	***	***
ApoE$^{-/-}$, M	***	***	***	***	**	N/A	***	***	***	***	***	***	***	***
MIX WT, F	ns	ns	ns	ns	***	***	N/A	ns	ns	*	ns	ns	***	***
MIX WT, M	ns	ns	ns	ns	***	***	ns	N/A	ns	ns	ns	ns	***	***
Cyp27a1$^{-/-}$ *Cyp46a1*$^{-/-}$, F	ns	ns	ns	ns	***	***	ns	ns	N/A	ns	ns	ns	***	***
Cyp27a1$^{-/-}$ *Cyp46a1*$^{-/-}$, M	**	ns	**	*	***	***	*	ns	ns	N/A	ns	ns	***	***
Cyp27a1$^{-/-}$ *Cyp46a1*$^{-/-}$ *Soat1*$^{-/-}$, F	ns	ns	ns	ns	***	***	ns	ns	ns	ns	N/A	ns	***	***
Cyp27a1$^{-/-}$ *Cyp46a1*$^{-/-}$ *Soat1*$^{-/-}$, M	ns	ns	ns	ns	***	***	ns	ns	ns	ns	ns	N/A	***	***
Cyp27a1$^{-/-}$ *Cyp46a1*$^{-/-}$ *ApoE*$^{-/-}$, F	**	***	**	**	***	***	***	***	***	***	***	***	N/A	ns
Cyp27a1$^{-/-}$ *Cyp46a1*$^{-/-}$ *ApoE*$^{-/-}$, M	***	***	***	***	***	***	***	***	***	***	***	***	ns	N/A

References

1. Wang, J.; Deretic, D. The arf and rab11 effector fip3 acts synergistically with asap1 to direct rabin8 in ciliary receptor targeting. *J. Cell. Sci.* **2015,** *128*, 1375-1385.
2. Wang, J.; Morita, Y.; Mazelova, J.; Deretic, D. The arf gap asap1 provides a platform to regulate arf4- and rab11-rab8-mediated ciliary receptor targeting. *EMBO J.* **2012,** *31*, 4057-4071.
3. Kizhatil, K.; Sandhu, N. K.; Peachey, N. S.; Bennett, V. Ankyrin-b is required for coordinated expression of beta-2-spectrin, the na/k-atpase and the na/ca exchanger in the inner segment of rod photoreceptors. *Exp. Eye Res.* **2009,** *88*, 57-64.
4. Pugacheva, E. N.; Jablonski, S. A.; Hartman, T. R.; Henske, E. P.; Golemis, E. A. Hef1-dependent aurora a activation induces disassembly of the primary cilium. *Cell* **2007,** *129*, 1351-1363.
5. Ran, J.; Yang, Y.; Li, D.; Liu, M.; Zhou, J. Deacetylation of alpha-tubulin and cortactin is required for hdac6 to trigger ciliary disassembly. *Sci. Rep.* **2015,** *5*, 12917.
6. Zhang, X.; Yuan, Z.; Zhang, Y.; Yong, S.; Salas-Burgos, A.; Koomen, J.; Olashaw, N.; Parsons, J. T.; Yang, X. J.; Dent, S. R., et al. Hdac6 modulates cell motility by altering the acetylation level of cortactin. *Mol. Cell* **2007,** *27*, 197-213.
7. Shah, N.; Ishii, M.; Brandon, C.; Ablonczy, Z.; Cai, J.; Liu, Y.; Chou, C. J.; Rohrer, B. Extracellular vesicle-mediated long-range communication in stressed retinal pigment epithelial cell monolayers. *Biochim. Biophys. Acta Mol. Basis. Dis.* **2018,** *1864*, 2610-2622.
8. Leyk, J.; Daly, C.; Janssen-Bienhold, U.; Kennedy, B. N.; Richter-Landsberg, C. Hdac6 inhibition by tubastatin a is protective against oxidative stress in a photoreceptor cell line and restores visual function in a zebrafish model of inherited blindness. *Cell Death Dis.* **2017,** *8*, e3028.
9. Yuan, H.; Li, H.; Yu, P.; Fan, Q.; Zhang, X.; Huang, W.; Shen, J.; Cui, Y.; Zhou, W. Involvement of hdac6 in ischaemia and reperfusion-induced rat retinal injury. *BMC Ophthalmol.* **2018,** *18*, 300.
10. Kim, T.; Loh, Y. P. Chromogranin a: A surprising link between granule biogenesis and hypertension. *J. Clin. Invest.* **2005,** *115*, 1711-1713.
11. Bechler, M. E.; Doody, A. M.; Ha, K. D.; Judson, B. L.; Chen, I.; Brown, W. J. The phospholipase a(2) enzyme complex pafah ib mediates endosomal membrane tubule formation and trafficking. *Mol. Biol. Cell* **2011,** *22*, 2348-2359.
12. Bechler, M. E.; de Figueiredo, P.; Brown, W. J. A pla1-2 punch regulates the golgi complex. *Trends Cell. Biol.* **2012,** *22*, 116-124.
13. Bechler, M. E.; Brown, W. J. Pafah ib phospholipase a2 subunits have distinct roles in maintaining golgi structure and function. *Biochim. Biophys. Acta* **2013,** *1831*, 595-601.
14. Balakrishnan, S. S.; Basu, U.; Shinde, D.; Thakur, R.; Jaiswal, M.; Raghu, P. Regulation of pi4p levels by pi4kiiialpha during g-protein-coupled plc signaling in drosophila photoreceptors. *J. Cell. Sci.* **2018,** *131*.
15. Zolyomi, A.; Zhao, X.; Downing, G. J.; Balla, T. Localization of two distinct type iii phosphatidylinositol 4-kinase enzyme mrnas in the rat. *Am. J. Physiol. Cell. Physiol.* **2000,** *278*, C914-920.
16. Wong, K.; Meyers dd, R.; Cantley, L. C. Subcellular locations of phosphatidylinositol 4-kinase isoforms. *J. Biol. Chem.* **1997,** *272*, 13236-13241.
17. Weixel, K. M.; Blumental-Perry, A.; Watkins, S. C.; Aridor, M.; Weisz, O. A. Distinct golgi populations of phosphatidylinositol 4-phosphate regulated by phosphatidylinositol 4-kinases. *J. Biol. Chem.* **2005,** *280*, 10501-10508.
18. Traikov, S.; Stange, C.; Wassmer, T.; Paul-Gilloteaux, P.; Salamero, J.; Raposo, G.; Hoflack, B. Septin6 and septin7 gtp binding proteins regulate ap-3- and escrt-dependent multivesicular body biogenesis. *PLoS One* **2014,** *9*, e109372.
19. Hu, J.; Bai, X.; Bowen, J. R.; Dolat, L.; Korobova, F.; Yu, W.; Baas, P. W.; Svitkina, T.; Gallo, G.; Spiliotis, E. T. Septin-driven coordination of actin and microtubule remodeling regulates the collateral branching of axons. *Curr. Biol.* **2012,** *22*, 1109-1115.
20. Hainzl, T.; Huang, S.; Sauer-Eriksson, A. E. Structure of the srp19 rna complex and implications for signal recognition particle assembly. *Nature* **2002,** *417*, 767-771.
21. Viotti, C. Er to golgi-dependent protein secretion: The conventional pathway. *Methods Mol. Biol.* **2016,** *1459*, 3-29.
22. Kim, H. R.; Kwon, M. S.; Lee, S.; Mun, Y.; Lee, K. S.; Kim, C. H.; Na, B. R.; Kim, B. N. R.; Piragyte, I.; Lee, H. S., et al. Tagln2 polymerizes g-actin in a low ionic state but blocks arp2/3-nucleated actin branching in physiological conditions. *Sci. Rep.* **2018,** *8*, 5503.
23. Na, B. R.; Kwon, M. S.; Chae, M. W.; Kim, H. R.; Kim, C. H.; Jun, C. D.; Park, Z. Y. Transgelin-2 in b-cells controls t-cell activation by stabilizing t cell - b cell conjugates. *PLoS One* **2016,** *11*, e0156429.

24. de las Heras, R.; Depaz, I.; Jaquet, V.; Kroon, P.; Wilce, P. A. Neuronal protein 22 colocalises with both the microtubule and microfilament cytoskeleton in neurite-like processes. *Brain Res.* **2007,** *1128*, 12-20.
25. Depaz, I. M.; Wilce, P. A. The novel cytoskeleton-associated protein neuronal protein 22: Elevated expression in the developing rat brain. *Brain Res.* **2006,** *1081*, 59-64.
26. Tajiri, Y.; Igarashi, T.; Li, D.; Mukai, K.; Suematsu, M.; Fukui, E.; Yoshizawa, M.; Matsumoto, H. Tubulointerstitial nephritis antigen-like 1 is expressed in the uterus and binds with integrins in decidualized endometrium during postimplantation in mice. *Biol. Reprod.* **2010,** *82*, 263-270.
27. Shen, M.; Jiang, Y. Z.; Wei, Y.; Ell, B.; Sheng, X.; Esposito, M.; Kang, J.; Hang, X.; Zheng, H.; Rowicki, M., et al. Tinagl1 suppresses triple-negative breast cancer progression and metastasis by simultaneously inhibiting integrin/fak and egfr signaling. *Cancer Cell* **2019,** *35*, 64-80 e67.
28. Yang, S. Y.; He, X. Y.; Schulz, H. Fatty acid oxidation in rat brain is limited by the low activity of 3-ketoacyl-coenzyme a thiolase. *J. Biol. Chem.* **1987,** *262*, 13027-13032.
29. Li, C. M.; Park, J. H.; He, X.; Levy, B.; Chen, F.; Arai, K.; Adler, D. A.; Disteche, C. M.; Koch, J.; Sandhoff, K., et al. The human acid ceramidase gene (asah): Structure, chromosomal location, mutation analysis, and expression. *Genomics* **1999,** *62*, 223-231.
30. Yu, F. P. S.; Sajdak, B. S.; Sikora, J.; Salmon, A. E.; Nagree, M. S.; Gurka, J.; Kassem, I. S.; Lipinski, D. M.; Carroll, J.; Medin, J. A. Acid ceramidase deficiency in mice leads to severe ocular pathology and visual impairment. *Am. J. Pathol.* **2019,** *189*, 320-338.
31. Sugano, E.; Edwards, G.; Saha, S.; Wilmott, L. A.; Grambergs, R. C.; Mondal, K.; Qi, H.; Stiles, M.; Tomita, H.; Mandal, N. Overexpression of acid ceramidase (asah1) protects retinal cells (arpe19) from oxidative stress. *J. Lipid Res.* **2019,** *60*, 30-43.
32. Marsillach, J.; Suzuki, S. M.; Richter, R. J.; McDonald, M. G.; Rademacher, P. M.; MacCoss, M. J.; Hsieh, E. J.; Rettie, A. E.; Furlong, C. E. Human valacyclovir hydrolase/biphenyl hydrolase-like protein is a highly efficient homocysteine thiolactonase. *PLoS One* **2014,** *9*, e110054.
33. Kumar, A.; Palfrey, H. A.; Pathak, R.; Kadowitz, P. J.; Gettys, T. W.; Murthy, S. N. The metabolism and significance of homocysteine in nutrition and health. *Nutr. Metab. (Lond)* **2017,** *14*, 78.
34. Adeva-Andany, M. M.; Gonzalez-Lucan, M.; Donapetry-Garcia, C.; Fernandez-Fernandez, C.; Ameneiros-Rodriguez, E. Glycogen metabolism in humans. *BBA Clin.* **2016,** *5*, 85-100.
35. Beerens, K.; Soetaert, W.; Desmet, T. Udp-hexose 4-epimerases: A view on structure, mechanism and substrate specificity. *Carbohydr. Res.* **2015,** *414*, 8-14.
36. Bar-Peled, L.; Sabatini, D. M. Regulation of mtorc1 by amino acids. *Trends Cell. Biol.* **2014,** *24*, 400-406.
37. Stacpoole, P. W. The pyruvate dehydrogenase complex as a therapeutic target for age-related diseases. *Aging Cell* **2012,** *11*, 371-377.
38. El-Hattab, A. W. Serine biosynthesis and transport defects. *Mol. Genet. Metab.* **2016,** *118*, 153-159.
39. Handley, R. R.; Reid, S. J.; Brauning, R.; Maclean, P.; Mears, E. R.; Fourie, I.; Patassini, S.; Cooper, G. J. S.; Rudiger, S. R.; McLaughlan, C. J., et al. Brain urea increase is an early huntington's disease pathogenic event observed in a prodromal transgenic sheep model and hd cases. *Proc. Natl. Acad. Sci. U. S. A.* **2017,** *114*, E11293-E11302.
40. Shayakul, C.; Clemencon, B.; Hediger, M. A. The urea transporter family (slc14): Physiological, pathological and structural aspects. *Mol. Aspects Med.* **2013,** *34*, 313-322.
41. Haider, N. B.; Mollema, N.; Gaule, M.; Yuan, Y.; Sachs, A. J.; Nystuen, A. M.; Naggert, J. K.; Nishina, P. M. Nr2e3-directed transcriptional regulation of genes involved in photoreceptor development and cell-type specific phototransduction. *Exp. Eye Res.* **2009,** *89*, 365-372.
42. Haider, N. B.; Jacobson, S. G.; Cideciyan, A. V.; Swiderski, R.; Streb, L. M.; Searby, C.; Beck, G.; Hockey, R.; Hanna, D. B.; Gorman, S., et al. Mutation of a nuclear receptor gene, nr2e3, causes enhanced s cone syndrome, a disorder of retinal cell fate. *Nat. Genet.* **2000,** *24*, 127-131.
43. Jia, L.; Oh, E. C.; Ng, L.; Srinivas, M.; Brooks, M.; Swaroop, A.; Forrest, D. Retinoid-related orphan nuclear receptor rorbeta is an early-acting factor in rod photoreceptor development. *Proc. Natl. Acad. Sci. U. S. A.* **2009,** *106*, 17534-17539.
44. Schaeren-Wiemers, N.; Andre, E.; Kapfhammer, J. P.; Becker-Andre, M. The expression pattern of the orphan nuclear receptor rorbeta in the developing and adult rat nervous system suggests a role in the processing of sensory information and in circadian rhythm. *Eur. J. Neurosci.* **1997,** *9*, 2687-2701.
45. Sun, W.; Kimura, H.; Hattori, N.; Tanaka, S.; Matsuyama, S.; Shiota, K. Proliferation related acidic leucine-rich protein pal31 functions as a caspase-3 inhibitor. *Biochem. Biophys. Res. Commun.* **2006,** *342*, 817-823.
46. Chemnitz, J.; Pieper, D.; Stich, L.; Schumacher, U.; Balabanov, S.; Spohn, M.; Grundhoff, A.; Steinkasserer, A.; Hauber, J.; Zinser, E. The acidic protein rich in leucines anp32b is an immunomodulator of inflammation in mice. *Sci. Rep.* **2019,** *9*, 4853.

47. Shen, S. M.; Yu, Y.; Wu, Y. L.; Cheng, J. K.; Wang, L. S.; Chen, G. Q. Downregulation of anp32b, a novel substrate of caspase-3, enhances caspase-3 activation and apoptosis induction in myeloid leukemic cells. *Carcinogenesis* **2010,** *31*, 419-426.
48. Jane-wit, D.; Surovtseva, Y. V.; Qin, L.; Li, G.; Liu, R.; Clark, P.; Manes, T. D.; Wang, C.; Kashgarian, M.; Kirkiles-Smith, N. C., et al. Complement membrane attack complexes activate noncanonical nf-kappab by forming an akt+ nik+ signalosome on rab5+ endosomes. *Proc. Natl. Acad. Sci. U. S. A.* **2015,** *112*, 9686-9691.
49. Herrmann, P.; Cowing, J. A.; Cristante, E.; Liyanage, S. E.; Ribeiro, J.; Duran, Y.; Abelleira Hervas, L.; Carvalho, L. S.; Bainbridge, J. W.; Luhmann, U. F., et al. Cd59a deficiency in mice leads to preferential innate immune activation in the retinal pigment epithelium-choroid with age. *Neurobiol. Aging* **2015,** *36*, 2637-2648.
50. Inafuku, S.; Klokman, G.; Connor, K. M. The alternative complement system mediates cell death in retinal ischemia reperfusion injury. *Front. Mol. Neurosci.* **2018,** *11*, 278.
51. Schnabolk, G.; Beon, M. K.; Tomlinson, S.; Rohrer, B. New insights on complement inhibitor cd59 in mouse laser-induced choroidal neovascularization: Mislocalization after injury and targeted delivery for protein replacement. *J. Ocul. Pharmacol. Ther.* **2017,** *33*, 400-411.
52. Faber, C.; Williams, J.; Juel, H. B.; Greenwood, J.; Nissen, M. H.; Moss, S. E. Complement factor h deficiency results in decreased neuroretinal expression of cd59a in aged mice. *Invest. Ophthalmol. Vis. Sci.* **2012,** *53*, 6324-6330.
53. Campbell, A. M.; Zagon, I. S.; McLaughlin, P. J. Astrocyte proliferation is regulated by the ogf-ogfr axis in vitro and in experimental autoimmune encephalomyelitis. *Brain Res. Bull.* **2013,** *90*, 43-51.
54. Hammer, L. A.; Zagon, I. S.; McLaughlin, P. J. Treatment of a relapse-remitting model of multiple sclerosis with opioid growth factor. *Brain Res. Bull.* **2013,** *98*, 122-131.
55. Dabo, S.; Maillard, P.; Collados Rodriguez, M.; Hansen, M. D.; Mazouz, S.; Bigot, D. J.; Tible, M.; Janvier, G.; Helynck, O.; Cassonnet, P., et al. Inhibition of the inflammatory response to stress by targeting interaction between pkr and its cellular activator pact. *Sci. Rep.* **2017,** *7*, 16129.
56. Stockley, R. A. The multiple facets of alpha-1-antitrypsin. *Ann. Transl. Med.* **2015,** *3*, 130.
57. Zhou, T.; Huang, Z.; Zhu, X.; Sun, X.; Liu, Y.; Cheng, B.; Li, M.; Liu, Y.; He, C.; Liu, X. Alpha-1 antitrypsin attenuates m1 microglia-mediated neuroinflammation in retinal degeneration. *Front. Immunol.* **2018,** *9*, 1202.
58. Ortiz, G.; Lopez, E. S.; Salica, J. P.; Potilinski, C.; Fernandez Acquier, M.; Chuluyan, E.; Gallo, J. E. Alpha-1-antitrypsin ameliorates inflammation and neurodegeneration in the diabetic mouse retina. *Exp Eye. Res.* **2018,** *174*, 29-39.
59. Mashima, R.; Saeki, K.; Aki, D.; Minoda, Y.; Takaki, H.; Sanada, T.; Kobayashi, T.; Aburatani, H.; Yamanashi, Y.; Yoshimura, A. Fln29, a novel interferon- and lps-inducible gene acting as a negative regulator of toll-like receptor signaling. *J. Biol. Chem.* **2005,** *280*, 41289-41297.
60. Sanada, T.; Takaesu, G.; Mashima, R.; Yoshida, R.; Kobayashi, T.; Yoshimura, A. Fln29 deficiency reveals its negative regulatory role in the toll-like receptor (tlr) and retinoic acid-inducible gene i (rig-i)-like helicase signaling pathway. *J. Biol. Chem.* **2008,** *283*, 33858-33864.
61. Hu, Y. H.; Zhang, Y.; Jiang, L. Q.; Wang, S.; Lei, C. Q.; Sun, M. S.; Shu, H. B.; Liu, Y. Wdfy1 mediates tlr3/4 signaling by recruiting trif. *EMBO Rep.* **2015,** *16*, 447-455.
62. Ning, F.; Li, X.; Yu, L.; Zhang, B.; Zhao, Y.; Liu, Y.; Zhao, B.; Shang, Y.; Hu, X. Hes1 attenuates type i ifn responses via vegf-c and wdfy1. *J. Exp. Med.* **2019,** *216*, 1396-1410.
63. Rosskothen-Kuhl, N.; Illing, R. B. Gap43 transcription modulation in the adult brain depends on sensory activity and synaptic cooperation. *PLoS One* **2014,** *9*, e92624.
64. Holahan, M. R. A shift from a pivotal to supporting role for the growth-associated protein (gap-43) in the coordination of axonal structural and functional plasticity. *Front Cell Neurosci* **2017,** *11*, 266.
65. Sato, Y.; Watanabe, N.; Fukushima, N.; Mita, S.; Hirata, T. Actin-independent behavior and membrane deformation exhibited by the four-transmembrane protein m6a. *PLoS One* **2011,** *6*, e26702.
66. Rosas, N. M.; Alvarez Julia, A.; Alzuri, S. E.; Frasch, A. C.; Fuchsova, B. Alanine scanning mutagenesis of the c-terminal cytosolic end of gpm6a identifies key residues essential for the formation of filopodia. *Front. Mol. Neurosci* **2018,** *11*, 314.
67. Honda, A.; Ito, Y.; Takahashi-Niki, K.; Matsushita, N.; Nozumi, M.; Tabata, H.; Takeuchi, K.; Igarashi, M. Extracellular signals induce glycoprotein m6a clustering of lipid rafts and associated signaling molecules. *J. Neurosci.* **2017,** *37*, 4046-4064.
68. Scorticati, C.; Formoso, K.; Frasch, A. C. Neuronal glycoprotein m6a induces filopodia formation via association with cholesterol-rich lipid rafts. *J. Neurochem.* **2011,** *119*, 521-531.
69. Formoso, K.; Garcia, M. D.; Frasch, A. C.; Scorticati, C. Evidence for a role of glycoprotein m6a in dendritic spine formation and synaptogenesis. *Mol. Cell. Neurosci.* **2016,** *77*, 95-104.

70. Rajalu, M.; Fritzius, T.; Adelfinger, L.; Jacquier, V.; Besseyrias, V.; Gassmann, M.; Bettler, B. Pharmacological characterization of gabab receptor subtypes assembled with auxiliary kctd subunits. *Neuropharmacology* **2015,** *88*, 145-154.
71. Seddik, R.; Jungblut, S. P.; Silander, O. K.; Rajalu, M.; Fritzius, T.; Besseyrias, V.; Jacquier, V.; Fakler, B.; Gassmann, M.; Bettler, B. Opposite effects of kctd subunit domains on gaba(b) receptor-mediated desensitization. *J. Biol. Chem.* **2012,** *287*, 39869-39877.
72. Perrin, L.; Lacas-Gervais, S.; Gilleron, J.; Ceppo, F.; Prodon, F.; Benmerah, A.; Tanti, J. F.; Cormont, M. Rab4b controls an early endosome sorting event by interacting with the gamma-subunit of the clathrin adaptor complex 1. *J. Cell. Sci.* **2013,** *126*, 4950-4962.
73. Krawczyk, M.; Leimgruber, E.; Seguin-Estevez, Q.; Dunand-Sauthier, I.; Barras, E.; Reith, W. Expression of rab4b, a protein governing endocytic recycling, is co-regulated with mhc class ii genes. *Nucleic Acids Res.* **2007,** *35*, 595-605.
74. Brown, T. C.; Correia, S. S.; Petrok, C. N.; Esteban, J. A. Functional compartmentalization of endosomal trafficking for the synaptic delivery of ampa receptors during long-term potentiation. *J. Neurosci.* **2007,** *27*, 13311-13315.
75. Kang, H. J.; Voleti, B.; Hajszan, T.; Rajkowska, G.; Stockmeier, C. A.; Licznerski, P.; Lepack, A.; Majik, M. S.; Jeong, L. S.; Banasr, M., et al. Decreased expression of synapse-related genes and loss of synapses in major depressive disorder. *Nat. Med.* **2012,** *18*, 1413-1417.
76. Mignogna, M. L.; D'Adamo, P. Critical importance of rab proteins for synaptic function. *Small GTPases* **2018,** *9*, 145-157.
77. Staropoli, J. F.; McDermott, C.; Martinat, C.; Schulman, B.; Demireva, E.; Abeliovich, A. Parkin is a component of an scf-like ubiquitin ligase complex and protects postmitotic neurons from kainate excitotoxicity. *Neuron* **2003,** *37*, 735-749.
78. Chen, Z. S.; Wong, A. K. Y.; Cheng, T. C.; Koon, A. C.; Chan, H. Y. E. Fipoq/fbxo33, a cullin-1-based ubiquitin ligase complex component modulates ubiquitination and solubility of polyglutamine disease protein. *J. Neurochem.* **2019,** *149*, 781-798.
79. Huang, Z.; Nie, L.; Xu, M.; Sun, X. H. Notch-induced e2a degradation requires chip and hsc70 as novel facilitators of ubiquitination. *Mol. Cell. Biol.* **2004,** *24*, 8951-8962.
80. Al-Ramahi, I.; Lam, Y. C.; Chen, H. K.; de Gouyon, B.; Zhang, M.; Perez, A. M.; Branco, J.; de Haro, M.; Patterson, C.; Zoghbi, H. Y., et al. Chip protects from the neurotoxicity of expanded and wild-type ataxin-1 and promotes their ubiquitination and degradation. *J. Biol. Chem.* **2006,** *281*, 26714-26724.
81. M., P. A.; N., S. J.; C., K. A.; G., C. S.; J., S. R.; C., L. D.; D., S. G.; BethAnn, M. Chip is an essential determinant of neuronal mitochondrial stress signaling. *Antioxidants & Redox Signaling* **2015,** *23*, 535-549.
82. Zhao, T.; Hong, Y.; Yin, P.; Li, S.; Li, X. J. Differential hspbp1 expression accounts for the greater vulnerability of neurons than astrocytes to misfolded proteins. *Proc. Natl. Acad. Sci. U. S. A.* **2017,** *114*, E7803-E7811.
83. Lee, J. S.; Seo, T. W.; Yi, J. H.; Shin, K. S.; Yoo, S. J. Chip has a protective role against oxidative stress-induced cell death through specific regulation of endonuclease g. *Cell Death Dis.* **2013,** *4*, e666.
84. Tian, Z.; D'Arcy, P.; Wang, X.; Ray, A.; Tai, Y. T.; Hu, Y.; Carrasco, R. D.; Richardson, P.; Linder, S.; Chauhan, D., et al. A novel small molecule inhibitor of deubiquitylating enzyme usp14 and uchl5 induces apoptosis in multiple myeloma and overcomes bortezomib resistance. *Blood* **2014,** *123*, 706-716.
85. Chitta, K.; Paulus, A.; Akhtar, S.; Blake, M. K.; Caulfield, T. R.; Novak, A. J.; Ansell, S. M.; Advani, P.; Ailawadhi, S.; Sher, T., et al. Targeted inhibition of the deubiquitinating enzymes, usp14 and uchl5, induces proteotoxic stress and apoptosis in waldenstrom macroglobulinaemia tumour cells. *Br. J. Haematol.* **2015,** *169*, 377-390.
86. Cai, J.; Xia, X.; Liao, Y.; Liu, N.; Guo, Z.; Chen, J.; Yang, L.; Long, H.; Yang, Q.; Zhang, X., et al. A novel deubiquitinase inhibitor b-ap15 triggers apoptosis in both androgen receptor-dependent and -independent prostate cancers. *Oncotarget* **2017,** *8*, 63232-63246.
87. Yun, D.; Zhuang, Y.; Kreutz, M. R.; Behnisch, T. The role of 19s proteasome associated deubiquitinases in activity-dependent hippocampal synaptic plasticity. *Neuropharmacology* **2018,** *133*, 354-365.
88. Kowalski, J. R.; Juo, P. The role of deubiquitinating enzymes in synaptic function and nervous system diseases. *Neural Plast.* **2012,** *2012*, 892749.
